# Antimicrobial and Photocatalytic Properties under Visible Blue LED Light of Silver Phosphate Supported on Biogenic Zeolite from Amazon Natural Source

**DOI:** 10.1021/acsomega.5c09905

**Published:** 2026-02-27

**Authors:** Ygor Geann dos Santos Leite, Francisco Xavier Nobre, José Fábio de Lima Nascimento, Wesley Victor De Sombra Quércia, Raiana Silveira Gurgel, Patrícia Melchionna Albuquerque, Yurimiler Leyet Ruiz, Ézio Sargentini-Júnior, Marcos A. Bolson, Yonny Romaguera-Barcelay, Ramón R. Peña-Garcia, Paulo Rogério da Costa Couceiro, Rosany Picolotto Carvalho

**Affiliations:** † Programa Multi-Institucional de Pós-Graduacão em Biotecnologia, Universidade Federal do Amazonas, Manaus, AM 69077-000, Brazil; ‡ Grupo de Recursos Energéticos e Nanomateriais (GREEN), Instituto Federal do Amazonas, Campus Manaus Centro (IFAM-CMC), Avenida Sete de Setembro, 1975, Manaus, AM 9020-120, Brazil; § Research Group on Chemistry Applied to Technology, School of Technology, Amazonas State University, Manaus 69050-020, Brazil; ∥ LPMAT - Department of Materials Engineering, 67892 Federal University of Amazonas, Manaus, AM 69077-000, Brazil; ⊥ Instituto Nacional de Pesquisas da Amazônia (INPA), Manaus, AM 69084-000, Brazil; # Universidade Federal Rural de Pernambuco, Programa de Pós-Graduação em Engenharia Física, Unidade Acadêmica do Cabo de Santo Agostinho, Recife, Pernambuco 54518-430, Brazil; ∇ Department of Chemistry, Federal University of Amazonas, Manaus, AM 69077-000, Brazil

## Abstract

In this study, we report the synthesis of silver phosphate supported on a phase mixture of Analcime and Pitiglianoite zeolites. The hybrid materials were obtained with AgP amounts of 25% (AgP_ZLT_25), 50% (AgP_ZLT_50), 75% (AgP_ZLT_75), and 95% (AgP_ZLT_95) relative to the ZLT mass. Structural characterization by X-ray diffraction (XRD) and structural refinement by the Rietveld method revealed that ZLT is composed of 71.02 ± 0.54% Analcime and 28.98 ± 0.47% Pitiglianoite zeolite. The percentage of AgP in the mixture, also quantified by the Rietveld method, resulted in values of 7.99 ± 0.46, 36.70 ± 4.84, 67.50 ± 5.06, and 93.74 ± 3.15% for the samples AgP_ZLT_25, AgP_ZLT_50, AgP_ZLT_75, and AgP_ZLT_95, respectively. Vibrational characterization by Raman and infrared (IR) spectroscopy revealed the presence of the main active vibrational modes of silver phosphate, silicate, and aluminate groups, in both pure and hybrid materials. The *E*_gap_ values revealed that ZLT effectively absorbs in the ultraviolet region, with *E*_gap_ equal to 3.65 eV. While the hybrid materials exhibited a decrease in the *E*_gap_ value, specifically between 3.63 eV (AgP_ZLT_25) and 2.35 eV (AgP_ZLT_95), in consequence of the contributions of the electronic transitions of AgP (*E*_gap_ = 2.35 eV). The photocatalytic performance of the materials prepared in the photodegradation of RhB dye in an aqueous medium under exposure to LED-simulated visible light resulted in dye discoloration percentages of 97.07% for the AgP_ZLT_95 sample and 93.56% for the AgP_ZLT_75 sample. Furthermore, analysis of the rate constant (*k*_app_) and half-life of the reactions (*t*_1/2_) revealed that the AgP_ZLT_95 catalyst was approximately 1,391.5 times more efficient compared to photolysis, exhibiting superior activity in the generation of superoxide radicals and vacancies. Additionally, it was found that zeolite acts as a sacrificial material, reducing the photocorrosion process of silver phosphate. Hybrid materials, as well as AgP, exhibited high antimicrobial activity, resulting in a minimum inhibitory concentration (MIC) of 0.01562 mg mL^–1^ for the bacteria *Staphylococcus aureus* and *Escherichia coli*, while for the fungi *Candida parapsilosis* and *Candida albicans*, the MIC was 0.03125 mg mL^–1^, as a result of redox processes involving reactive oxygen species (ROS), as well as silver-ion activity and [AgO_4_] and [PO_4_] clusters in the cell wall and internal structures of microorganisms.

## Graphical abstract

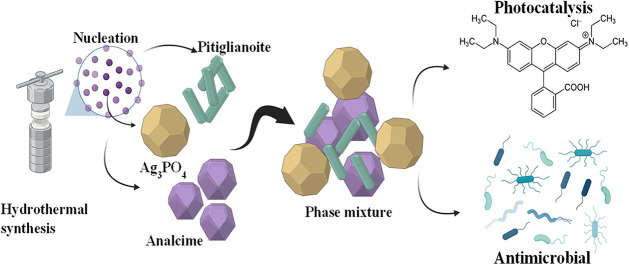

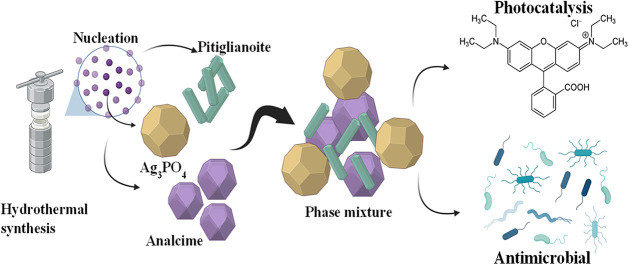

## 1 Introduction

Metal nanoparticles and semiconductor oxides based on silver, copper, nickel, cobalt, zinc, platinum, and gold have been studied in relation to microbial inhibition, cancer cell treatment, electrochemical sensor applications, and the remediation of effluents containing POAs.^1–3^ Therefore, scientific reports confirm the efficient activity of silver-based compounds in the inhibition of multidrug-resistant strains of bacteria and fungi, especially relating to the semiconductor silver phosphates (Ag_3_PO_4_), silver tungstates (Ag_2_WO_4_), silver molybdates (Ag_2_MoO_4_), silver vanades (AgVO_3_), silver chromates (Ag_2_CrO_4_), zeolites doped with silver ions, and titanium oxide decorated with silver ions (Ag@TiO_2_).^4–6^

Botelho et al.^7^ report the photocatalytic and antimicrobial performance of silver phosphate against strains of *Staphylococcus aureus* methicillin-resistant was investigated in detail, where different factors favorable to the addition of photocatalytic and antimicrobial properties are raised, where crystalline defects, distortions of Ag–O and P–O bonds, particle size, and morphology, contribute significantly to the properties performed. In addition, Takeno et al.^8^ investigated the photocatalytic and antimicrobial performance of microcrystals of silver phosphates synthesized by solvothermic and hydrothermal processing, focusing on the influence of a mixture of distilled water, acetone, ammonium hydroxide, and isopropyl alcohol as a polarity modifier of the solutions. Therefore, it was observed that when preparing the solutions, as well as the processing of the suspensions obtained with the mixture of distilled water and acetone, there was the formation of the tetrapoid morphology for the silver phosphate microcrystals, with high exposure of the {110} surface plane, which performed high photocatalytic performance in the discoloration of the synthetic dye rhodamine B (RhB) in aqueous medium, as well as antimicrobial activity against different strains of multidrug-resistant microorganisms.

Silver phosphate (AgP) is a semiconductor of the type *p* which exhibits a cubic crystal structure and a high quantum yield, due to the effective absorption of photons in the visible spectrum region, mainly at wavelengths equal to or less than 530 nm, associated with those with optical bandgap energy (*E*_gap_), determined experimentally to be close to 2.43 eV.^9^ It is commonly obtained by the chemical synthesis routes, mainly the chemical precipitation method,^10^ sol–gel method,^11^ hydrothermal and solvothermic processing,^8^ sonochemical route,^12^ and microwave-assisted hydrothermal method.^13^ Each of these approaches has particularities that imply modifications to structural, optical, and morphological characteristics, which ultimately contribute to the intended applications to a greater or lesser degree. The literature^8,14–16^ has revealed that silver phosphate with preferential exposure of the {110} face, especially microcrystals with tetrapod-shaped morphology, exhibit optimized photocatalytic and antimicrobial performance due to the higher surface energy for this configuration, which favors greater interaction between the s orbitals of the elements silver, oxygen, and phosphorus in the driving bank, which are the frontier for the reduction processes, as well as the contribution of the species photogenerated in the Valence Band (VB), which have a predominant contribution of the Ag 4d, O2py, and 2pz orbitals.

These reported characteristics allow the use of AgP in different applications, highlighting the heterogeneous photocatalysis of different persistent organic pollutants in aqueous medium under natural or simulated visible light,^17^ development of photoelectrochemical sensors,^18^ bactericidal and fungicidal applications,^19^ and treatment of cancer cells.^20^

However, silver phosphate has as disadvantages, the costs of obtaining, considering that silver nitrate is an analytical reagent of more added value compared to other transition metals such as copper, nickel and zinc, as well as the high rate of photocorrosion, a phenomenon in which the catalyst loses efficiency of catalytic performance over different cycles of radiation exposure, due to the decomposition of the primary structure, caused by the high leaching rate of silver ions into the reaction medium.^9,21^

To mitigate the photocorrosion effect of AgP, recent literature has focused on developing materials with enhanced optical and morphological properties through chemical doping of the AgP structure as well as combining this material with other inorganic structures that are resistant to photocorrosion processes, resulting in hybrid materials known as heterojunctions. Among the materials reported in the literature, used as a coupling to AgP, are titanium oxide (TiO_2_),^22^ zinc oxide (ZnO),^22^ graphitic carbon nitride (g-C_3_N_4_),^23^ bismuth phosphate (BiPO_4_),^24^ reduced graphene oxide (rGO),^25^ cobalt phosphate (Co_3_(PO_4_)_2_),^26^ silver carbonate (Ag_2_CO_3_),^27^ cadmium sulfide (CdS),^28^ silver molybdate,^29^ and cerium oxide (CeO_2_).^30^ The combination of silver phosphate with natural clay has also been investigated, with emphasis on the study carried out by Teixeira et al.,^31^ who combined AgP with montmorillonite clay and fibrous sepiolite, as well as the study by Ma et al.,^32^ which immobilized AgP crystals in the bentonite clay, in both cases, attributed photocatalytic properties to the hybrid materials under lower photocorrosion rate.

In Brazil, in addition to its rich biodiversity, mineral wealth is highlighted, especially concerning the natural deposits of clay minerals that are little known or explored.^33^ In Amazonas, although it is known worldwide for having one of the most extensive preserved or little explored forests on the planet, where it is home to a particular amount of species that make up the fauna and flora of the region, it is also the stage for interests directly or indirectly related to the deposits of gold, tin, niobium, rare metals and Kaolinite.^17,34^ In addition, the disposal of significant amounts of aquatic organisms and residual biomass derived from fruit extraction has been the subject of studies for technological applications.

In view of the importance of heterogeneous catalysts and micro and macroporous materials for various purposes, there is a growing interest in obtaining materials classified as zeolites, mainly using alternative synthesis routes to commercial reagents, which introduce clay minerals, such as kaolin,^35^ glass powder,^19^ and freshwater sponges,^36^ and fly ash as substitutes for analytical-grade aluminum oxide and silicon sources. Therefore, zeolites are basically composed of the three-dimensional organization of clusters [SiO_4_] Silicon Oxide Tetrahedral and Clusters [AlO_4_] tetrahedral of aluminum oxide in cage-shaped arrangements, which contain cations such as Na^+^, K^+^, H^+^, Ca^2+^, and Ba^2+^ as counterions of the structure, as well as water molecules, resulting in the empirical formula *M*_*y*_^*z*+^(*Si*_1–*x*_ *Al*_*x*_ *O*_2_)^*x*−^, where *x* = *yz* is often limited to 0 ≤ *x* ≤ 0.5.^31^ There are appreciable amounts of natural and synthetic zeolites that differ in chemical composition, pore volume, and size, and cluster organization [SiO_4_] and [AlO_4_] in the composition of the crystalline lattice. Therefore, pure and modified zeolites have been widely applied for various purposes, which include the catalysis of biofuels,^37^ petroleum cracking,^38^ vegetable and mineral oil bleachers,^39^ remediation of effluents containing heavy metals,^40^ persistent organic pollutants,^41^ as well as electrochemical sensors, supercapacitors,^42^ controlled-release systems,^38^ and drug excipients.

Based on the information presented, and supported by the work developed by Lacerda et al.,^36,39^ and in the work developed by Takeno et al.,^8^ the present study investigated the photocatalytic properties of silver phosphate immobilized in zeolite obtained from Metakaolin extracted from Amazonian Kaolinite, together with biogenic silica extracted from the freshwater sponge *Metania Kiliani*. The materials were characterized using X-ray diffraction, infrared vibrational spectroscopy, and energy-dispersive X-ray spectroscopy (EDX), and optical properties were characterized by spectrophotometry in the UV–vis region, employing the diffuse reflectance technique (UV–vis DRS) and colorimetric analysis. In addition, the performance of the materials obtained in the discoloration of solutions containing the dye RhB in aqueous medium was investigated, as well as antimicrobial activity against multidrug-resistant strains of the *Escherichia coli*, *S. aureus*, *C. albicans*, and *Candida parapsilosis*, as shown by the promising results available throughout the following topics of this study.

## 2 Materials and Methods

### 2.1 Preparation of Silicon Oxide from a Biogenic Source

Samples of Cauxi (*Metania Kiliani*) were collected on the desposites of the Rio Negro, in the municipality of Iranduba, Amazonas, under geographic coordinates 3°5′29.630″ south latitude and 60°26′25.559″ west longitude. The freshwater sponge was collected from fallen tree trunks on the riverbanks, packed in clean and properly labeled plastic bags, and later stored in transport boxes before being taken to the laboratory.

The production of biogenic silica was conducted by the drying of the cauxi samples collected in the sun, under natural conditions characteristic of the tropical climate of the Amazon region, in May 2024, in the absence of rainfall, for a period until the complete removal of moisture was ensured. Then, the dried material was meticulously crushed until it reached a fine spicule state. Precisely 20 g of the crushed spicules were then subjected to an oxidizing solution composed of hydrogen peroxide (H_2_O_2_), nitric acid (HNO_3_), and distilled water in a 8:7:1 ratio (v/v/v). This mixture was heated at 90 °C for 20 min. Subsequently, the material was collected by filtration using a porcelain filter, and this step was systematically repeated until the precipitate reached a white color, indicating complete purification. Finally, the purified material was subjected to drying at 160 °C for 2 h, resulting in biogenic silica from Cauxi. Figure S1a,b, available in the Supporting Information, shows the X-ray diffraction pattern, as well as the elemental analysis by X-ray fluorescence (XRF) of the biogenic silica prepared.

### 2.2 Obtention of Natural Metakaolin from Amazon Kaolinite Clay

The natural kaolin samples were collected in the municipality of Presidente Figueiredo, Amazonas, more precisely at Km 64 of BR 174, with geographic coordinates 2°24′51.9″S 60°01′56.66″W. The collected material was initially sifted, using the material passed through a 200-mesh sieve. Subsequently, 100 g of the previously processed mineral was inserted into a 200 mL porcelain crucible and subjected to heat treatment in an air atmosphere in a muffle furnace at a heating rate of 10 °C min^–1^ until it reached 650 °C for 6 h. The material was cooled and later characterized to confirm the chemical transformation of the crystal lattice. Figure S2a,b, available in the Supporting Information, shows the X-ray diffraction (XRD) patterns of kaolinite and metakaolinite.

### 2.3 Zeolite Synthesis Using a Natural Source of Amazonian Clay

The synthesis of zeolite from mineral and biogenic sources was performed by preparing a suspension identified as A, containing 3.81 g of metakaolin, along with 0.585 g of sodium hydroxide and 10 mL of distilled water, in a 50 mL plastic beaker. On the other hand, another beaker with the same characteristics was used in the preparation of a suspension identified as B, where 3.84 g of biogenic silica was dispersed in 10 mL of distilled water, along with 0.585 g of sodium hydroxide. After the total dispersion of both suspensions, suspension A was added to suspension B under constant magnetic agitation at 450 rpm, at room temperature, and the mixture was maintained under the same conditions for 30 min. After 30 min, the resulting suspension was kept at rest at room temperature for 24 h, which was then followed by hydrothermal processing at 150 °C for 48 h in a Teflon reactor (capacity of 100 mL), lined in stainless steel, which was introduced into a circulating air oven and heated at a rate of 10 °C min^–1^. The material was collected by centrifugation using plastic tubes with a capacity of 15 mL, with 4000 rpm cycles for 5 min. In each cycle, the supernatant was discarded and distilled water was added to perform the subsequent cycle. The washing cycles were concluded when the pH of the supernatant remained close to 8. The precipitate was dried in a circulating air oven at 85 °C for 48 h, until the constancy of the mass contained in the tubes was observed, confirming the complete elimination of the water content present. This material was stored in a sterile plastic bottle for further characterization and use as a support material in subsequent syntheses and is referred to, for all intents and purposes, as the ZLT sample.

### 2.4 Preparation of Supported Silver Phosphate Immobilized on Zeolite

The synthesis of pure silver phosphate (AgP) was performed following the steps previously described in the previous study.^29^ Thus, 1 mmol of silver nitrate (AgNO_3_) 40 mL of water:acetone solution was dissolved in a 50:50 (v/v) ratio, contained in a Falcon tube (50 mL capacity), dispersed with the aid of a Vortex shaker (Ika Instruments, Brazil), while in another Falcon tube of the same volume, 3 mmol of dibasic sodium phosphate was dissolved with the aid of a vortex shaker. After total solubilization, the solution containing the phosphate ions (PO_4_^3–^), was transferred to a 100 mL reactor cup and subjected to constant magnetic agitation. On the other hand, the solution containing silver ions (Ag^+^) was added drop by drop, resulting in a rapid color change to the characteristic strong yellow tone of silver phosphate. The system was heated to a constant temperature of 120 °C for 12 h. After the hydrothermal treatment, the material was cooled to room temperature, and the precipitate was collected by centrifugation, using cycles with a speed of 10,000 rpm for 5 min. The supernatant was discarded after each wash, and in the final cycle, the material was washed with acetone. The precipitate was dried in an incubator at 65 °C for 6 h until a constant mass of the material was achieved, and this sample was designated as AgP. The reactions involved in the formation of silver phosphate are presented in eq 1.

1 $$3{\mathrm{AgNO}}_{3}\left(\mathrm{aq}\right)+{\mathrm{Na}}_{2}{\mathrm{HPO}}_{4}\cdot 2H_{2}O\left(\mathrm{aq}\right)\overset{H_{2}O}{\to }{\mathrm{Ag}}_{3}{\mathrm{PO}}_{4}\left(s\right)+3N{O_{3}}^{-}\left(\mathrm{aq}\right)+2{\mathrm{Na}}^{+}\left(\mathrm{aq}\right)+H^{+}\left(\mathrm{aq}\right)$$

Adopting the approach described in the previous paragraphs for the AgP sample, the supported materials were prepared by adding the percentages of zeolite synthesized in the steps described above to the solution to be subjected to the hydrothermal process. Therefore, stoichiometric amounts of ZLT were added to the reactor cup along with 50 mL of the solution containing phosphate ions, which remained under constant magnetic agitation. The solution containing silver ions was then added dropwise until a total transfer was achieved. Then, the system was closed and subjected to heat treatment at 120 °C for 12 h. The material obtained in each synthesis was collected by centrifugation, using the same conditions as those for washing the AgP sample, and dried in an oven at 65 °C for 6 h. The samples were prepared in the intended proportions of 25, 50, 75, and 95% (w/w), resulting in samples coded as AgP_ZLT_25, AgP_ZLT_50, AgP_ZLT_75, and AgP_ZLT_95, respectively. To schematically represent the steps adopted and described in the previous paragraphs, a schematic representation is provided in Figure 1, where the steps of synthesis of pure silver phosphate (AgP) are described, as well as the materials supported in zeolite (ZLT).

**Figure 1:**
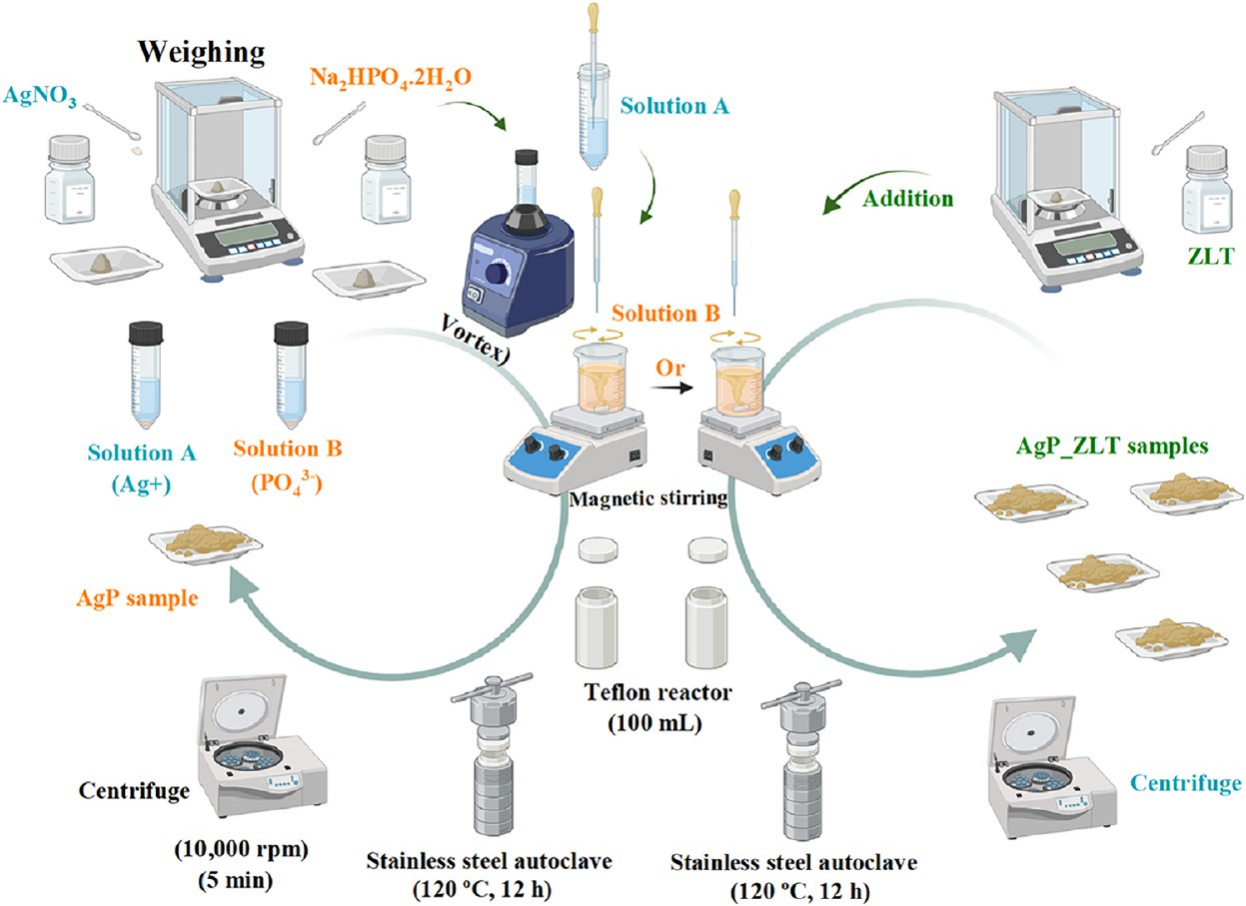
Schematic representation of AgP and AgP_ZLT materials.

### 2.5 Characterization

#### 2.4.1 X-ray Diffraction and Rietveld Refinement

The diffraction patterns of the samples were collected using the powder method, operating Shimadzu XRD7000 equipment with a copper anode as the source of X-ray radiation (Cu Kα= 0.15406 nm), and 2θ interval between 10 and 100° with a step of 0.02° min^–1^. On the other hand, the structural refinement was performed using the Rietveld method, which computes the crystallographic information with the aid of the FullProf software, version January 2025, for Windows.

#### 2.4.2 Vibrational Infrared Spectroscopy

The vibrational spectra of the prepared samples were collected using an Agilent spectrometer, model Cary 630 FTIR, with spectra obtained using the attenuated total reflectance (ATR) method. The measurements were performed in the range of 650 to 4000 cm^–1^ in percentage transmittance mode, utilizing 32 scans with a resolution of 4 cm^–1^.

#### 2.4.3 Scanning Electron Microscopy (SEM)

The morphology and dimensions of the particles that compose the prepared materials were analyzed using scanning electron microscopy (SEM), with an FEI Company equipment model Quanta FEG 250 operating at an acceleration voltage of 10 kV, and a range of 1 to 30 kV. Micrographs were collected by initially dispersing 20 mg of the sample in 1 mL of acetone, which was subsequently subjected to ultrasonic stirring for 3 min, followed by the removal of 100 μL of the suspension with the aid of a single-channel pipet and then dripped onto the carbon tape that was already covering the aluminum substrate (stub). The samples were metallized with gold by sputtering using the quantum metallizer, model Q150R ES. SEM micrographs were performed in two detectors (secondary SE and backscattered BE).

#### 2.4.4 Raman Vibrational Spectroscopy

The Raman vibrational spectra were collected using a Horiba confocal Raman spectrometer, model Xplora Plus, equipped with a charge-coupled device (CCD) system, which excited the samples with a red laser at a wavelength of 785 nm. The spectra were recorded in the interval of 80 to 1100 cm^–1^, using 50% of the maximum laser power, adopting an integration time of 5 s, 5 coadditions, and 3 accumulations, adjusted in the LabSpec 6 software.

#### 2.4.5 X-ray Fluorescence Spectroscopy

The semiquantitative analysis was performed using Malvern Panasonic equipment, model EPSON 4, coupled with a high-resolution silicon deviation detector (SDD) of 135 eV and Mn–Kα radiation, using the loose powder method. Therefore, for the assay performed in triplicate, approximately 5 g of sample was accommodated in a polyethylene cup that already contained a Mylar polymeric membrane (polyethylene, Kapton).

#### 2.4.6 Analytical Measurement of Silver by ICP-OES

Analytical analyses of Ag^+^ ions were performed by inductively coupled plasma atomic emission spectrometry (ICP-OES) using a Thermo iCAP-7600 DUO instrument, employing an ultrasonic nebulizer and autosampler from CETAC, models ASX 520 and U5000AT+, with argon, purity >99.9999% (White Martins-Manaus, AM, Brazil), also used for plasma generation, nebulization, and as an auxiliary gas. For external mobility, solutions were prepared from a 1000 mg L^–1^ stock solution with successive dilutions, obtaining a linear range of 0.5 to 10 ppm of Ag^+^ ions and monitoring the peak intensity at a wavelength of 328.06 nm, characteristic of the silver emission line. The concentration curve, the spectra that generated the curve, and the results obtained are available in Figure S3a–c, available in the Supporting Information.

#### 2.4.7 Morphological Analysis by Transmission Electron Microscopy (TEM)

The morphological analysis of the nanoparticles was performed by transmission electron microscopy using a Jeol microscope, model JEM-1400Flash, operating at a voltage of 80 kV and with a magnification of 1,500,000 times. The samples were initially dispersed in ultrapure water by a washer machine for 3 min and subsequently deposited on a copper grid with a carbon film of standard thickness and mesh size of 300 mesh (Sigma-Aldrich).

#### 2.4.8 Photocatalytic Assay

The photocatalytic tests were carried out using a handmade system consisting of an acrylic box with dimensions of 20 × 20 × 15 cm, containing on one side a microfan with dimensions of 8 × 8 × 3.8 cm, current of 68 mA, power of 15.2 W and rotation speed of 3150 rpm, powered by a 12 V and 1.5 A source, with a voltage of 127 V. In addition, in the upper layer, nine LEDs with a wavelength of 425 nm (royal blue), a voltage of 3.0–3.4 V, a current of 600–700 mA, and a luminous flux of 50 Lumens each, are connected in parallel. They form three rows of 3 LEDs, distributed over an area of 10 cm^2^. The oxygenation of the system was achieved through an aeration pump with a flow rate of 1.8 L min^–1^, while a magnetic stirrer promoted the stirring of the system. Therefore, for each assay, 50 mg of the sample to be tested was used, along with 50 mL of Rhodamine B (RhB) dye solution, inserted into a beaker with a capacity of 250 mL, which was initially dispersed in an ultrasonic bath, using an ultrasonic washer with a power of 160 W and a frequency of 42 kHz, for 10 min at room temperature, to achieve the adsorption balance of the dye on the catalyst. The photocatalytic performance was investigated by monitoring the wavelength of maximum absorption of the dye, in this case, at 554 nm, at consecutive time intervals of 2 min, with the time of −10 min being the solution before the adsorption equilibrium and the time of 0 min representing the sample after reaching adsorption equilibrium. The aliquots were collected until reaching a maximum time of 10 min, where about 2 mL was collected at consecutive intervals of 2 min, centrifuging them at 10,000 rpm for 3 min, and examining the supernatant that was introduced into quartz cuvettes, with a capacity of 2.5 mL, followed by the collection of the dye spectrum in the range of 190 to 640 nm, in absorbance module, using a Shimadzu spectrophotometer, model UV1200.

#### 2.4.9 Electrochemistry Study

The electrochemical assays were performed using cyclic voltammetry (*C–V*) and electrochemical impedance spectroscopy (EIS) in an electrolyte solution of 0.5 mol L^–1^ potassium hydroxide. To this end, the preparation of the working electrodes consisted of preparing a paste containing the active material (zeolites), carbon *black* (conductive agent), and polyvinylidene fluoride polymer, PVDF (binder), in a mass ratio of 80:10:10. The paste was dispersed in the solvent *N*-methylpyrrolidone (NMP), then deposited on a glass carbon electrode as substrate, with a geometric area of 0.3 diameter, which acted as a current collector. The application was performed with the aid of a micropipette, ensuring uniform distribution on the GCE surface. Subsequently, the solvent was evaporated in a circulating air oven at 65 °C for 1 h.

Electrochemical measurements were performed using Autolab equipment, model PGSTAT 101 Potentiostat/Galvanostat. For all electrochemical tests, an electrochemical cell with a conventional three-electrode system was used, featuring a platinum wire as the counter electrode, a Ag/AgCl (3 M KCl) reference electrode, and films of the doped and pure zeolites as the working electrode.

The VC analysis was adopted to characterize the electrochemical processes in a sweep speed range of 100 mVs^–1^. At the same time, the electrochemical impedance (ESI) technique was used in the analysis of the electrochemical phenomena at the interfaces between the electrode and the support electrolyte, using the same electrolyte used in the *C–V* tests, adopting the frequency range of 0.1 to 1 × 10^5^ Hz, amplitude of 0.005 V_RSM_, Sine wave type, and 10 frequencies per decade.

### 2.6 Antimicrobial Assays

#### 2.5.1 In Vitro Susceptibility Assay

The microdilution technique was used.^40,41^ The samples were tested against commercially acquired strains from Cefar Diagnóstica Ltd.: *E. coli* (CCCD-E005), *S. aureus* (CCCD-S009), *Candida albicans* (CCCD-CC001), and *C. parapsilosis* (CCCD-CC004). The assay was performed using a sterile 96-well microplate where, in triplicate, 100 μL of microbial inoculum at a concentration of 1.5 × 10^4^ CFU mL^–1^ (fungi) and 5 × 10^5^ CFU mL^–1^ (bacteria) was inserted, along with 100 μL of the sample to be tested at a concentration of 1 mg mL^–1^ (solubilized with sterile deionized water followed by approximately 15 min in an ultrasonic bath). Successive dilutions were performed to determine the minimum inhibitory concentration (MIC), which is the lowest concentration capable of inhibiting microbial growth. The positive control used was Terbinafine 0.2 mg mL^–1^ for the antifungal tests and Levofloxacin 0.125 mg mL^–1^ for the antibacterial tests. As a negative control, the microbial inoculum was inserted along with sterile deionized water (used for solubilization and dilutions), and for sterility control, 100 μL of the sterile culture medium used to prepare the inoculum was used (Sabouraud broth for fungi and Mueller Hinton broth for bacteria). Subsequently, the plates were sealed and incubated in a BOD (biochemical oxygen demand) incubator at 37 °C for 24 h (bacteria) and 48 h (fungi). After this period, the absorbances were measured in a microplate reader (Loccus LMR-96), at wavelengths of 630 nm for the tests with bacteria and 540 nm for the tests with yeasts, and compared to the negative control.

Based on the determined MICs, an assay was performed to evaluate the contact time curve of antimicrobial inhibition.^42^ Samples were prepared at a concentration of 62.5 μg mL^–1^ for testing against *E. coli* and *S. aureus*; at a concentration of 31.2 μg mL^–1^ for tests with *C. albicans*; and at 15.6 μg mL^–1^ for tests with *C. parapsilosis*. Positive controls were also prepared at the same concentration as the samples for comparison purposes. Absorbance measurements of the suspensions were taken at 0, 1, 3, 6, 12, and 24 h for the bacterial assays and at 0, 1, 3, 6, 12, 24, 36, and 48 h for the yeast assays.

## 3 Results and Discussion

### 3.1 X-ray Diffraction and Structural Refinement by the Rietveld Method

As shown in Figure 1Sa,b, available in the Supporting Information, the structural characterization of the biogenic silica obtained from the freshwater sponge, i.e., from Cauxi, presents, predominantly, a characteristic graphic profile of amorphous silicon oxide, i.e., absence of intense and well-defined peaks in the interval 2θ between 5 and 90°. However, peaks in the 2θ values were identified = 21.12, 26.66, 36.84, 39.65, 50.25, 60.03, 64.18, 68.39, 77.79, and 76.52°, which confirm the presence of crystalline silicon oxide in the form of quartz, and confirm the crystallographic information contained in the Inorganic Crystal Structure Database (ICSD) card no. 200722, as well as the literature consulted.^43^ In Figure 1Sb, the elemental analysis by X-ray fluorescence (XRF) is presented, where the composition of the sample, performed in triplicate, reveals the predominance of the element silicon, accounting for 82.43%, followed by aluminum, at 7.17%. In contrast, the respective percentages for the element’s phosphorus, sulfur, potassium, calcium, and titanium were 0.76, 0.715, 0.339, 0.457, and 0.62%. In a study conducted by Lacerda et al.,^39^ it is reported that biogenic silica was obtained from the spicules of the Amazonian Cauxi using leaching and bleaching processes with an acid solution composed of aqua regia. As part of the analyses performed, a purely amorphous diffraction pattern was obtained for biogenic silica with no crystallographic planes associated with quartz or other oxides. On the other hand, Ribeiro et al.,^44^ performed the structural, morphological characterization and semiquantitative analysis by energy-dispersive X-ray (EDX) of specimens of freshwater sponge from the Amazon, collected in the extension of the Rio Negro, in the vicinity of the municipality of Manaus, Amazonas, and obtained diffraction standard for the material calcined at 550 °C with crystallographic planes characteristic of quartz, as well as iron silicate hydroxide and an amorphous profile, which was associated with the presence of amorphous silica.

The transformation of Kaolinite into Metakaolinite was also accompanied by X-ray diffraction, as can be seen in the diffraction patterns available in Figure S2a,b, available in the Supporting Information. In Figure S2a, it is possible to observe that the profile and intensity of the diffraction planes contained in the interval 2θ between 10° and 80° are characteristic of materials with considerable crystallinity and organization at short and long ranges.^45^ From the indexation of the diffraction peaks, it was possible to verify the majority presence of the mineral aluminum silicate hydroxide, popularly known as Kaolinite, which has a chemical formula Al_2_Si_2_O_5_(OH)_4_, which has Anorthic crystal structure with space group *C1* and lattice parameters *a* = 5.1400Å, *b* = 8.9100 Å, and *c* = 7.2600 Å and angles α, β, and γ, with respective values of 91.67, 104.67, and 90°. This structure also has a unit cell volume of 160.75 Å^3^ and two formulas per unit cell, exhibiting high similarity with the crystallographic information contained in the ICSD card number. 200723, as well as the diffraction patterns reported in the literature.^46–48^ Additionally, the presence of diffraction planes associated with the hexagonal structure of silicon oxide (quartz) was confirmed, in agreement with the information contained in ICSD card no. 20593, which has lattice parameters *a* = *b* = 4.8691 Å and *c* = 5.3703 Å, with a unit cell volume 110.26 Å^3^ and three formulas per unit cell. On the other hand, the diffraction pattern collected for Metakaolinite (Figure S3b) reveals that the graphic profile indicates the presence of materials with structural disorder, in this specific case, characteristic of aluminum and silicon oxides, resulting from the structural disorganization of Kaolinite to obtain Metakaolinite. This effect is the result of the transformation of the three-dimensional structure, which occurred under a heat treatment temperature close to 650 °C, which eliminates the water molecules from the structure, as well as breaking the bonds between the [SiO_4_] and [AlO_4_] clusters of tetrahedral geometry, agreeing with the observations made by Soares et al.^49^ who found that this endothermic event occurs at a temperature of maximum mass loss at 525 °C, as presented in the thermogravimetric analysis correlated with X-ray diffraction. In addition, as shown in Figure S2b, the presence of diffraction peaks associated with the tetragonal structure of anatase was found (TiO_2_), which has a space group *I*41*/amd* and network parameters *a* = *b* = 3.7800 Å, and *c* = 9.5100 Å, with unit cell volume of 135.88 Å^3^ and four formulas per unit cell, all of this information, in excellent agreement with ICSD card no. 200392 and the literature consulted.^50–52^ The occurrence of titanium dioxide in the composition of Metakaolinte is commonly reported in the literature for clay minerals from the Amazon region, especially when they undergo processing by heat treatment, where the intensity of the crystallographic planes of the Kaolinite structure is reduced due to the conversion, and consequent structural change, into amorphous oxides and quartz, which favors the proportional increase in the intensity of the crystallographic planes of anatase.^53–55^

The structural characterization was performed by X-ray diffraction, as shown in Figure 2a–d, as well as the structural refinement graphs presented in Figure 3a–f. From the indexing of the diffraction patterns presented in Figure 2a, it was possible to identify the presence of two distinct crystalline structures that comprise the ZLT sample. Therefore, indexing all plans in the range 2θ between 10° and 100°, the presence of zeolite, popularly known as Analcime,^56^ i.e., sodium aluminum catena-disilicate hydrate, with the chemical formula NaAl­(Si_2_O_6_) (H_2_O). This mineral exhibit tetragonal space group structure *Ia*3̅*d*, with lattice parameters *a* = *b* = 13.728(1) Å and *c* = 13.722(1) Å and unit cell volume (*V*) equal to 2586.02(42) Å^3^, and has 16 formulas per unit cell (*Z* = 16), according to the information contained in the Inorganic Crystal Structure Database (ICSD) no. 34878. On the other hand, there was also indexation with the structure of the zeolite Faujasite-Na; however, due to the absence of crystallographic cards associated with Faujasite-Na in the X’Pert HighScore Plus database, it was decided to use the crystallographic information on its isomorph, in this case, the mineral Pitiglianoite.^51^ This mineral exhibits a hexagonal structure, with the chemical formula Na_6_K_2_(Al_6_Si_6_O_24_) (SO_4_)­(H_2_O)_2_, space group *P*63, with lattice parameters *a* = *b* = 22.1210 Å and *c* = 5.2210 Å, unit cell volume equivalent to 2212.55 Å^3,^ and three formulas per unit cell as per ICSD card no. 66463. For the AgP sample, all crystallographic planes indexed within the described interval corroborate the crystallographic information contained in ICSD card no. 201361, referring to silver phosphate, with the chemical formula Ag_3_PO_4_, which has a cubic space group structure *P*4̅3*n*, and network parameters *a* = *b* = *c* = 6.0130 Å and two formulas per unit cell.^14,21^

**Figure 2:**
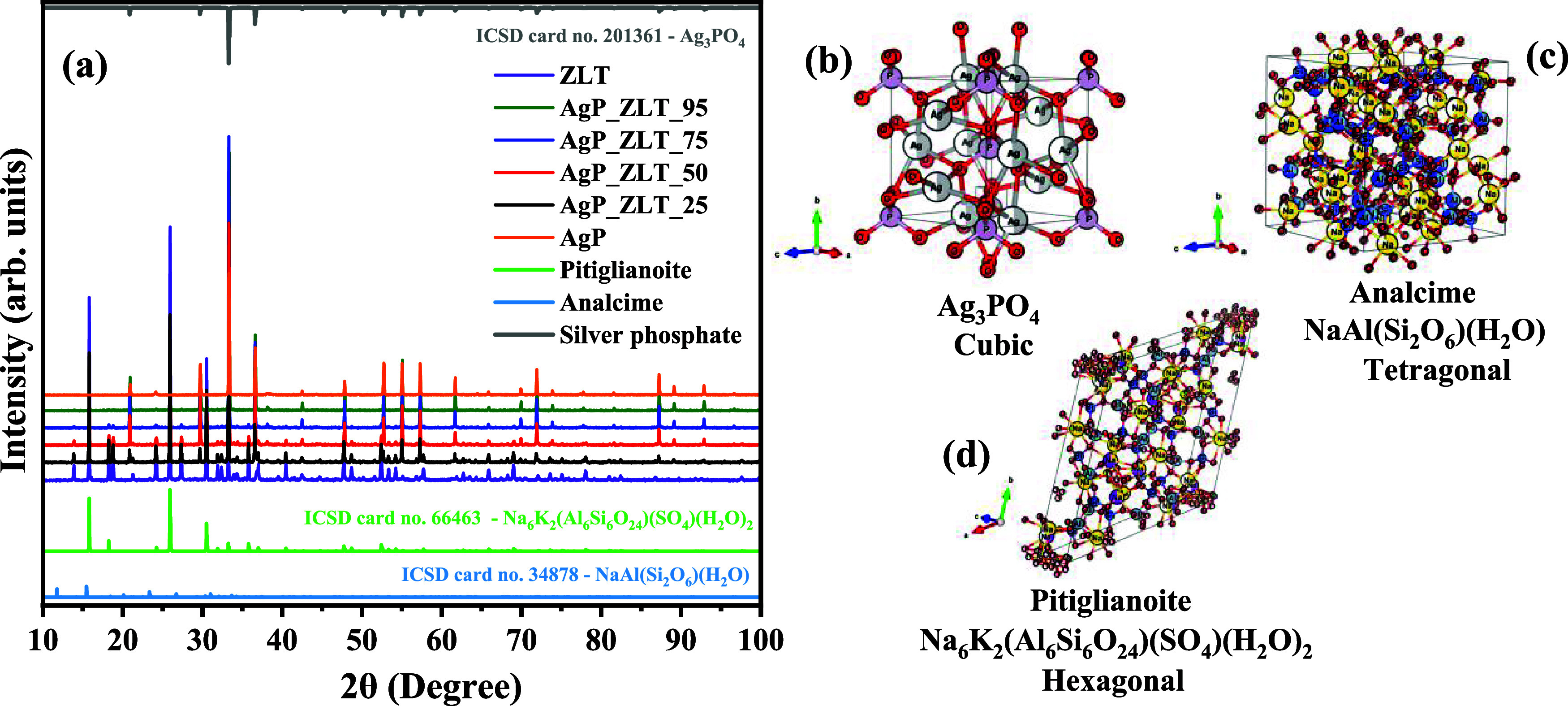
(a) XRD diffraction pattern of ZLT, AgP, and supported zeolites (AgP_ZLT) for different proportions and schematic representation for the unit cell of (b) silver phosphate, (c) analcime zeolite, and (d) Pitiglianoite zeolite.

**Figure 3:**
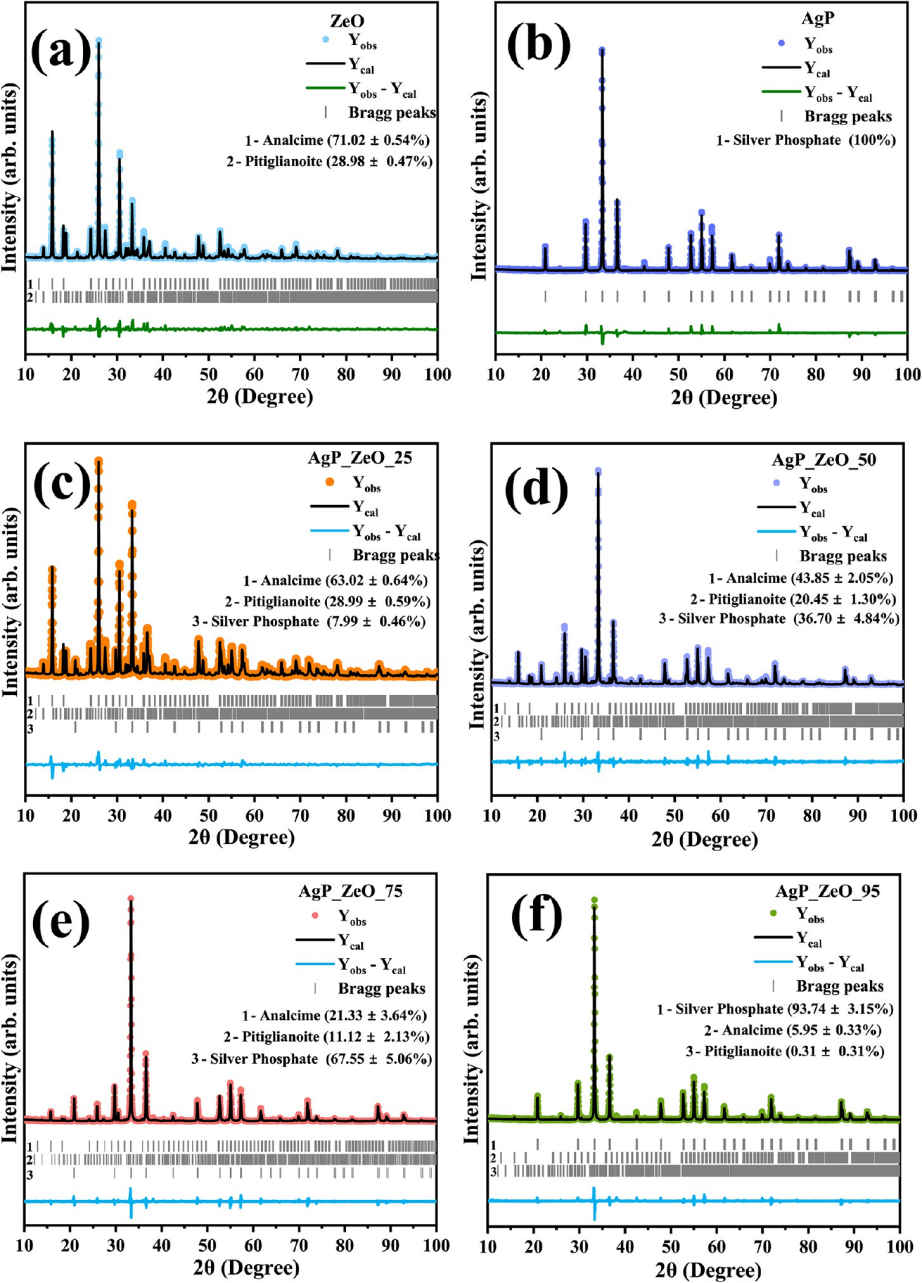
Rietveld refinement plot for (a) ZLT, (b) AgP, (c) AgP_ZLT_25, (d) AgP_ZLT_50, (e) AgP_ZLT_75, and (f) AgP_ZLT_95 samples.

The schematic representation of the unit cell presented for silver phosphate (Figure 2b), zeolite analcime (Figure 2c), and zeolite Pitiglianoite (Figure 2d) was created from the crystallographic information contained in their respective crystallographic cards using the Visualization for Electronic and Structural Analysis (VESTA) software, version 3.90.3a, for Windows. Therefore, it is possible to notice that the cubic structure of silver phosphate has clusters of tetrahedral symmetry for the units [AgO_4_] and [PO_4_]. On the other hand, the visualization of the unit cell of the zeolite Analcime reveals that the three-dimensional organization is composed of clusters [SiO_4_] and [AlO_4_] tetrahedral symmetry, while tetrahedral clusters [NaO_6_] exhibit distorted octahedral symmetry. Finally, the zeolite Pitiglianoite exhibits clusters with geometry similar to that observed for the zeolite Analcime, where the units [SiO_4_] and [AlO_4_] have tetrahedral symmetry, while clusters [NaO_6_] exhibit distorted octahedral symmetry.

A detailed study of the crystallographic information was conducted using structural refinement by the Rietveld method, as illustrated graphically in Figure 3a–f, as well as summarized in Table 1. In this study, the structural refinement of the samples was carried out, using the FullProf software, version December 2023, adopting the Thompson-Cox-Hastings pseudo-Voigt * Axial divergence asymmetry function for the adjustment of the intensity and profile of the diffraction peaks.^57^ In addition, the polynomial function was used with 6 orders for the initial adjustment of the background, while for the last refinement cycle, the linear interpolation model was adopted, introducing the background of the diffraction pattern extracted by the WinPlotr tool, available in the software itself. The computation quality of the refined data was monitored by the *R* quality indicators, where values close to 2 for the chi-square are considered statistically reproducible and reliable, especially for materials that exhibit single phase.^58^ Among other information, the network parameters were refined (*a*, *b* and *c*, α, β, and γ), occupancy factor (*O*_cc_), atomic coordinates (*x*, *y*, and *z*), phase composition (χ_r_), unit cell volume (*V*), background, and parameters *U*, *V*, *W*, *IG*, *X*, and *Y*, associated with the Caglioti function.

**Table 1:** Rietveld Refinement Results for AgP, ZLT, AgP_ZLT_25, AgP_ZLT_50, AgP_ZLT_75, and AgP_ZLT_95 Samples and Crystallographic Information of ICSD Cards^a^

	**samples**						**ICSD card references**		
**parameters**	AgP	ZLT	AgP_ZLT_25	AgP_ZLT_50	AgP_ZLT_75	AgP_ZLT_95	⬢	⧫	⬕
Ag_3_ PO_4_									
*a = b = c* (Å)	6.012(8)		6.013(1)	6.013(6)	6.013(9)	6.014(5)	6.011(1)		
α = β = γ (°)	90		90	90	90	90	90		
*V* (Å^3^)	217.389(1)		217.408(9)	217.473(3)	217.508(1)	217.571(3)	217.1(9)		
χ_r_	100		7.99(0.46)	36.70(4.84)	67.55(5.06)	93.74(3.15)			
*D*_hkl_ (nm)	85		49	70	60	65			
Analcime									
*a* = *b* (Å)		13.723(2)	13.720(9)	13.727(6)	13.732(9)	13.733(5)		13.728(1)	
*c* (Å)		13.7226	13.7474	13.7506	13.7516	13.7608		13.722(1)	
α = β = γ (°)		90	90	90	90	90		90.000	
*V* (Å^3^)		2584.315(7)	2588.131(1)	2591.263(4)	2593.442(6)	2595.424(6)		2586.02(42)	
χ_r_ (%)		71.02(0.54)	63.02(0.64)	42.85(2.05)	21.33(1.15)	5.95(0.15)			
*D*_hkl_ (nm)		43	59	61	58	65			
Pitiglianoite									
*a* = *b* (Å)		22.067(1)	22.102(7)	22.124(8)	22.136(8)	22.141(5)			22.121(3)
*c* (Å)		5.214(3)	5.211(2)	5.213(9)	5.218(6)	5.199(1)			5.221(1)
α = β (°)		90	90	90	90	90			90.000
γ (°)		120	120	120	120	120			120.000
*V* (Å^3^)		2198.955(3)	2204.757(6)	2210.294(7)	2214.698(5)	2207.338(1)			2212.55(73)
χ_r_ (%)		28.98(0.47)	28.99(0.59)	20.45(1.30)	11.12(0.55)	0.31(0.025)			
*D*_hkl_ (nm)		37	35	38	37	70			

a Legend: ⬢ = Ag_3_PO_4_, ICSD card no. 201361; ⧫ = NaAl­(Si_2_O_6_)­(H_2_O), ICSD card no. 34878; ⬕ = Na_6_K_2_(Al_6_Si_6_O_24_)­(SO_4_)­(H_2_O)_2_, ICSD card no. 66463.

As shown in Figure 3a, it is possible to notice that the structural refinement performed for the ZLT sample resulted in excellent agreement for the experimental data (*Y*_obs_) and computed data (*Y*_cal_), where it is clearly possible to notice that the residual line (*Y*_obs_–*Y*_cal_) presents few differences, which indicates that there was a high correlation of the profile and intensity of the diffraction planes between the theoretical model adopted for the experimental data. In addition, it is confirmed that the phase composition for the present structures consists of 71.02 ± 0.54% of the zeolite Analcime, while for the zeolite Pitiglianoite, the percentage is 28.98 ± 0.47%. The analysis of the network parameters for the zeolite Analcime reveals that the length of the shafts *a* = *b* = *c* was 13.723(2) Å, while the unit cell volume was 2584.315(7) Å^3^, while for the zeolite Pitiglianoite, they were *a* = 22.067(1) Å, *b* = 22.067(1) Å, and *c* = 5.214(3) Å, with unit cell volume of 2198.955(3) Å^3^. The structural refinement performed for silver phosphate, as shown in Figure 3b, confirmed the presence of silver phosphate as a single phase, which resulted in the computation of the data performed at the length of the axes *a* = *b* = *c* = 6.0128 Å, with a unit cell volume of 217.3891 Å^3^.

The samples containing silver phosphate supported in the zeolite matrix, specifically AgP_ZLT_25, AgP_ZLT_50, AgP_ZLT_75, and AgP_ZLT_95, were refined and are graphically presented in Figure 3c–f, respectively. Although the expected composition of silver phosphate in the samples should be 25, 50, 75 and 95% in relation to the composition of the fraction corresponding to ZLT, the percentages were actually 7.99 ± 0.46, 36.70 ± 4.84, 67.55 ± 5.06, and 93.74 ± 3.15%, respectively. The difference between the theoretical and experimentally obtained values is due to the dynamism of the hydrothermal processing as well as the ion exchange effect that can occur during the synthesis of silver phosphate in the presence of zeolite, which reduces the reactivity between Ag^+^ and PO_4_^–^ ions.

The results also reveal that the composition between the phases of zeolite Analcime and zeolite Pitiglianoite differ when comparing the percentages present in the ZLT sample and the percentages obtained in the AgP_ZLT_25, AgP_ZLT_50, AgP_ZLT_75, and AgP_ZLT_95 samples, confirming the dynamism in the hydrothermal process adopted, which results in modifications related to the conversion of one phase into another, as well as bond distortions, variation in crystallite size, length, and angle between chemical bonds.

The size of crystallite (*D*_hkl_) of the crystal structures present in the samples was calculated adopting the Scherrer model,^57^ as presented in eq 2.

2 $${\mathrm{D̅}}_{\mathrm{hkl}}=\frac{k\lambda }{\beta_{\mathrm{Tot}}}$$

where *k* corresponds to the constant associated with the shape of the particles, λ is the wavelength of the radiation used in the diffractometer in the acquisition of crystallographic data, while β_Tot_ is the half-height width of the diffraction peaks (fwhm), subtracted from the instrumental contributions on the line width of the crystallographic planes. In this case, β_Tot_ was obtained from eq 3.

3 $$\beta_{\mathrm{Tot}}=\sqrt{\beta_{\mathrm{sample}}^{2}-\beta_{\mathrm{instrument}}^{2}}$$

Therefore, in this study, we used the Rietveld refinement of the diffraction standard of lanthanum hexaboride, LaB_6_ (Sigma-Aldrich, purity >99.0%) to obtain the values of β_instrument_, which was automatically computed by the FullProf software, using the Instrumental Resolution File (.IRF).

From the data obtained and presented in Table 1, it is possible to notice that the crystallite size for the crystals that compose the AgP sample was 34 nm and that when supported on the zeolite, in obtaining the samples AgP_ZLT_25, AgP_ZLT_50, AgP_ZLT_75, and AgP_ZLT_95 via hydrothermal processing, It resulted in the sizes of 59, 61, 58, and 65 nm, respectively. These values are consistent with those reported in the literature,^59^ in particular, the study carried out by Mirsalari and Nezamzadeh-Ejhieh^28^ reports the obtaining of silver phosphate microcrystals by the method of chemical precipitation at room temperature, using 5 h of magnetic agitation, which obtained materials with considerable crystallinity and crystallite size of 25.36 nm. On the other hand, in the study reported by Bozetine, Boukennous, and Moudir,^54^ Ultrafine silver phosphate powders were synthesized by the chemical precipitation method, resulting in crystallite sizes for the materials obtained between 73 and 91 nm, with a notable size dependence observed depending on the adopted precursor.

In this study, sodium phosphate dihydrate was used as the precursor together with silver nitrate. At the same time, the synthesis method was the conventional solvothermic method, which suffers a strong effect of the pressure and polarity of the solvents involved, i.e., acetone and distilled water, which leads to the dynamics and nucleation, maturation, and growth of the crystals to provide homogeneity and high crystallinity for the prepared materials. Thus, the variation in crystallite size observed in this study may be related to the process of anchoring the particles on the surface of the zeolite, which may lead to preferences of crystallographic planes, deformations of the Ag–O and P–O bonds in the clusters [PO_4_] and [AgO_4_], oxygen vacancies, and crystalline defects, during the crystal maturation process.^15,27,55,60^

The vibrational characterization was performed by using Raman spectroscopy and infrared spectroscopy, as shown in Figure 4a,b. The literature reports that silver phosphate with a cubic structure and space group *P*4̅3*n* has 18 active modes in Raman spectroscopy and exhibits the following irreducible formula:

4 $$\Gamma_{\mathrm{Raman}}=2A_{1}+4E+12T_{2}$$

where the symmetry modes A_1_ are the symmetrical stretch modes that are one-dimensional with respect to the axis of highest order for the symmetry system, while the E modes are the doubly degenerate, i.e., two-dimensional modes, which involve the angular vibrations, and the symmetry element T_2_ indicates the vibrational modes that move perpendicular to the axis of most excellent order, i.e., bending movements.

**Figure 4:**
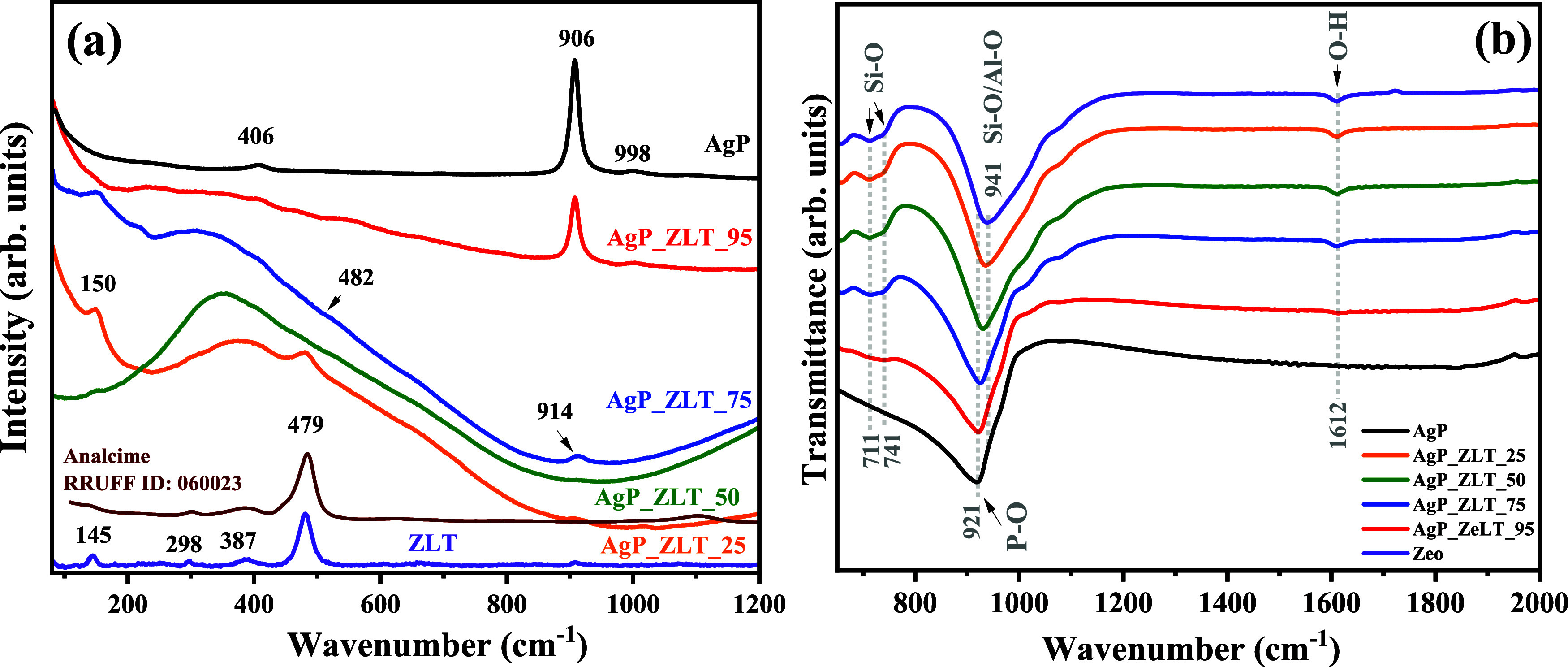
Vibrational (a) Raman spectra and (b) infrared spectra of AgP, ZLT, AgP_ZLT_25, AgP_ZLT_50, AgP_ZLT_75, and AgP_ZLT_95.

As can be seen in the pure silver phosphate spectrum, available in Figure 4a, three main vibrational modes were identified in the vibrational spectrum in the range between 80 and 1200 cm^–1^, these being, in the values of 406 cm^–1^ (E), 906 cm^–1^ (A_1_) and 998 cm^–1^ (T_2_), associated with symmetrical inflections of the groups [PO_4_], symmetrical elongation of O–P–O bonds and asymmetric elongation of O–P–O bonds.^61^ The absence of the other vibrational modes in the spectrum of the AgP sample may be related to the method of synthesis or the resolution of the equipment used in the data acquisition, as reported in the literature.^62^ It is possible to notice that the band of higher intensity, related to the symmetrical elongation of O–P–O bonds, suffers a reduction in intensity with the decrease of the percentage of silver phosphate in the mixture, as can be observed in the spectra of samples AgP_ZLT_95 and AgP_ZLT_75, being practically absent in the spectra obtained for samples AgP_ZLT_50 and AgP_ZLT_5. This behavior can be explained by the synergistic effect of the luminescent properties of zeolite Analcime, which can undergo chemical doping by silver ions, as observed in the reduction of the silver phosphate content in AgP_ZLT25 and AgP_ZLT-50 samples, previously discussed in the analysis by X-ray diffraction and structural refinement using the Rietveld method. This behavior of synergistic effect of the luminescent properties between zeolites and metallic nanoparticles or ion exchange has been reported in the literature,^63,64^ especially involving silver ions and silver nanoparticles, in addition to being favorable to ion exchange with the cations present in zeolitic structures, which can form clusters of the type Ag_n_^m+^ (0 ≤ m ≤ n).

From the Raman spectrum of pure zeolite (ZLT), it is possible to verify the presence of two main bands in the interval between 80 and 1200 cm^–1^, more precisely in the values of 145, 298, 387, and 479 cm^–1^. These bands are characteristic of zeolite analcime, according to the standard spectrum of the mineral collected in Dalla, Oregon, which is available in the American Mineralogist Crystal Structure Database (https://rruff.info/). Therefore, these vibration bands are due to the stretching of the Si–O and Al–O bonds present in the clusters [SiO_4_] and [AlO_4_] of tetrahedral symmetry.^65^ These bands are consistent with the bands reported in the study conducted by Tian et al.,^56^ which involved synthesizing pure zeolite Analcime doped with silver ions using the hydrothermal method at 155 °C for 12 h of reaction.

The vibrational spectra in the infrared region are shown in Figure 4b, where it is possible to identify the band of strong intensity centered on 921 cm^–1^, which is due to the asymmetric stretches of the P–O links, present in the clusters [PO_4_] of tetrahedral symmetry.^7^ In addition, the vibrational spectrum for pure zeolite, i.e., ZLT sample, exhibits an intense band situated at 921 cm^–1^, characteristic of zeolytic materials, and which is due to the asymmetric stretches of the T-O-T bonds, where T = Si or Al, as reported by Novembre and Gimeno,^18^ while the bands centered on 711 and 741 cm^–1^ are due to the torsional movements of the T-O-T connections.^19^ It is noticeable that as the silver phosphate content in the composition increases from the AgP_ZLT_25 sample to the AgP_ZLT_95 sample, the bands present in 711 and 741 cm^–1^ lose intensity, as well as the displacement of the maximum to the band associated with the symmetrical stretching of the Si–O–Si/Al–O–Al bonds, as a result of the contributions of the interactions between the structures.

The optical characterization of pure zeolite, pure silver phosphate, and heterojunctions was characterized by UV–vis spectroscopy using diffuse reflectance, as shown in Figure 5, and in the Tauc graphs available in Figure 6a–f, which allowed for the estimation of the band gap value (*E*_gap_) of the prepared materials.

**Figure 5:**
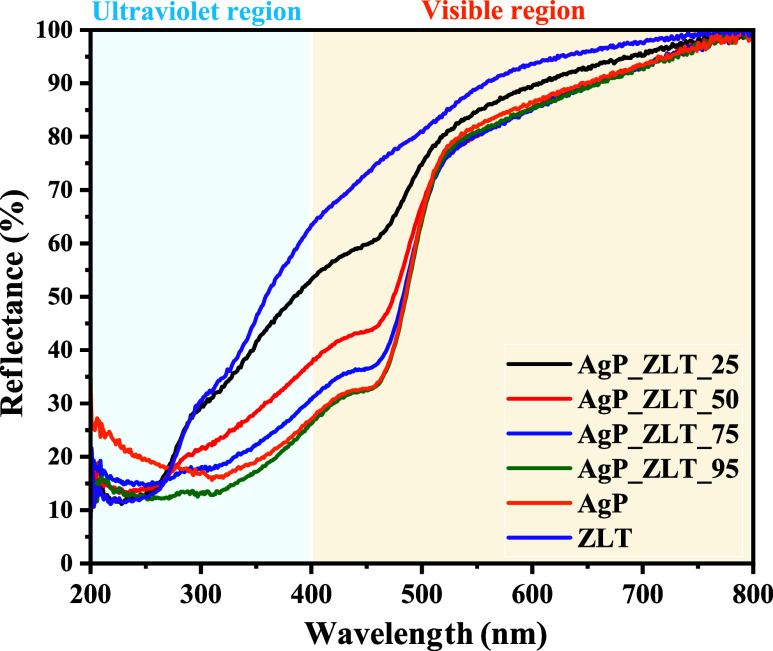
DRS spectra of AgP, ZLT, AgP_ZLT_25, AgP_ZLT_50, AgP_ZLT_75, and AgP_ZLT_95 samples.

**Figure 6:**
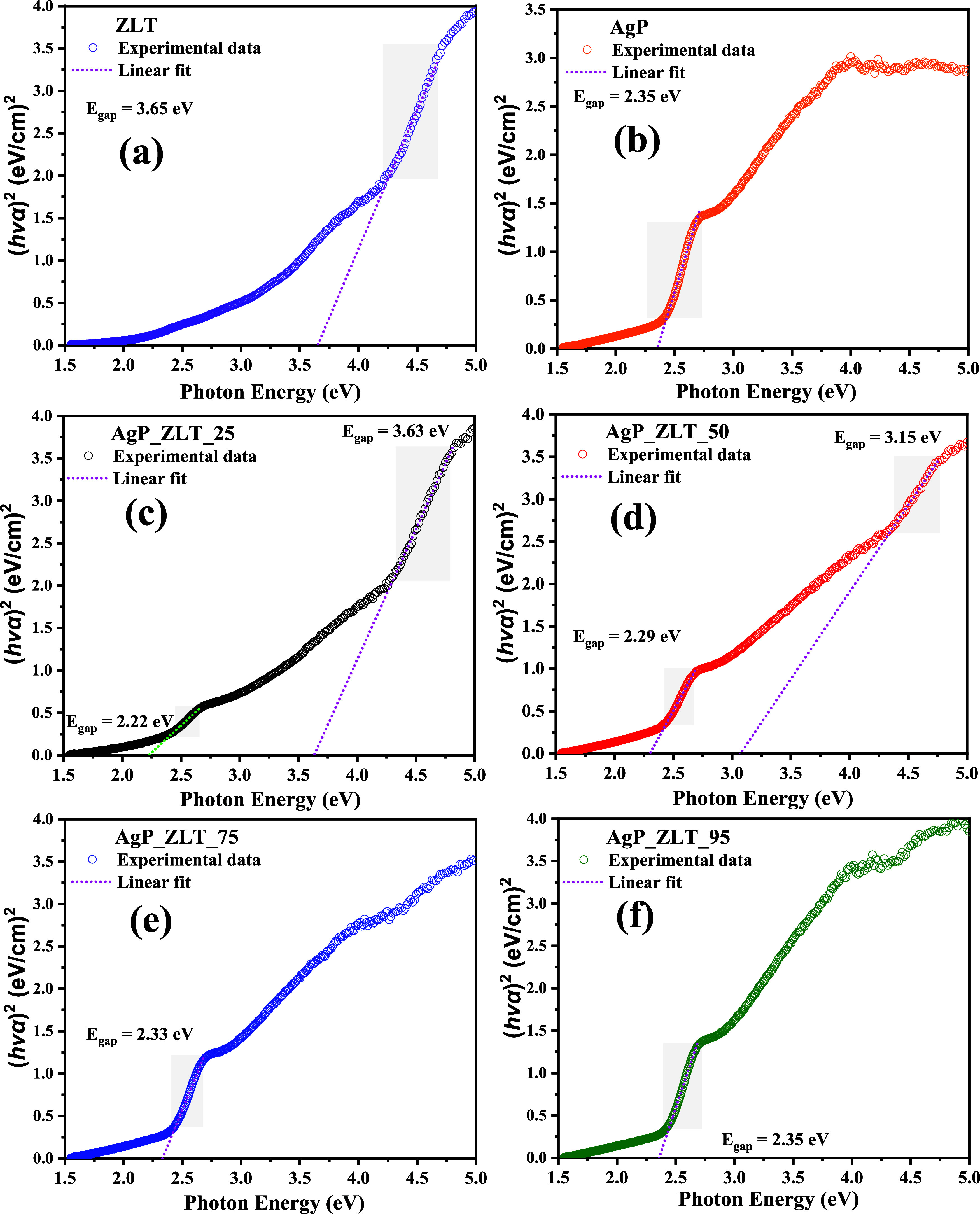
Tauc plot of (a) ZLT, (b) AgP, (c) AgP_ZLT_25, (d) AgP_ZLT_50, (e) AgP_ZLT_75, and (f) AgP_ZLT_95 to estimate the optical band gap.

As shown in Figure 5, the spectra of the materials collected in the range of 200 to 800 nm exhibit different optical characteristics, resulting in varying percentage reflectance values for each region of the electromagnetic spectrum.^66^ Therefore, it is possible to highlight that silver phosphate characteristically has little absorption of photons at wavelengths greater than 500 nm. However, it will present strong absorption, that is, a reduction in the percentage of reflectance, at wavelengths below 480 nm, a characteristic behavior of semiconductors.^67^ This is due to the electronic transitions involving the semiconductor bands, specifically the valence band (VB) and the conduction band (CB), where electrons present in the valence band, upon absorbing photons, are excited to the conduction band. The energy necessary for this promotion is a characteristic of the semiconductor, which can be correlated with specific factors, including particle size, composition of crystalline phases, presence of crystalline defects or doping ions, as well as oxygen vacancies.^67^

The graphic profile for pure silver phosphate (AgP) is observed categorically in samples containing silver phosphate in their composition, i.e., AgP_ZLT_25, AgP_ZLT_50, AgP_ZLT_75, and AgP_ZLT_95 samples, corroborating the discussions made on the topic of X-ray diffraction (XRD) and structural refinement by the Rietveld method. However, for the ZLT sample, this profile of the curve for the visible spectrum region is not observed, which confirms that these optical properties observed for the combined materials are due to the optical contributions of the electronic transitions involving the energy bands of silver phosphate. In addition, a rapid decrease in the percentage reflectance values is noted for the region with wavelengths below 400 nm, which indicates that zeolite Analcime and Pitiglianoite have photon absorption predominantly in the ultraviolet B region.^68^

The value of the *E*_gap_ for pure silver phosphate, pure zeolite, and mixtures was estimated by initially converting the wavelength values to energy, using the modified Planck equation, as presented in eq 5.^29^

5 $$E_{gap}=\frac{1240\;eV}{\lambda }$$

where λ is the wavelength of the radiation, in this case, limited to the range between 200 and 800 nm. On the other hand, the values of percentage reflectance (*R*%), in the values of the molar absorptivity coefficient (*k*′) and spreading coefficient (*s*′), using the mathematical formulas presented respectively in eqs 6 and 7.^69^

6 $$k^{\prime }={\left(1-\frac{R\%}{100}\right)}^{2}$$

7 $$s^{\prime }=2\times \frac{R\%}{100}$$

From the information obtained by eqs 6 and 7, the values of the linear absorption coefficient (α′) were calculated as presented in eq 8. The *E*_gap_ was then estimated for each sample by the Tauc method, extrapolating the straight section of the paraboloid curve obtained to the values of (α′*h*ν)*^n^* vs photon energy (*h*ν), mathematically represented in eq 9.^62–64^

8 $$\alpha^{\prime }=\frac{k^{\prime }}{s^{\prime }}$$

9 $${\left(\alpha^{\prime }h\nu \right)}^{1/n}=A_{1}\left(h\nu -E_{\mathrm{gap}}\right)$$

where A_1_ is the constant of proportionality, *h* is Planck’s constant (*h* = 6.63 × 10^–34^ Js^–1^), ν is the frequency, and *n* corresponds to the type of electronic transition, which can assume values of *n* = 0.5 for direct transitions allowed, *n* = 2 for permitted indirect transitions, and *n* = 1.5 for prohibited direct transitions, according to the computational calculations performed by Trench et al.^61^ In this case, adopting the theory of functional density (DFT) and direct allowable electron transitions (*n* = 2), it was confirmed that electron transitions related to the cubic structure of silver phosphate have a contribution mostly from the Ag 4d orbitals as well as O2py and O2pz in the conduction band (CB). On the other hand, in the valence band (VB), there is a predominance of contributions involving the s orbitals of the elements silver, phosphorus, and oxygen.

As can be seen in Figure 6a–f, the values of *E*_gap_ estimated from Tauc’s plot, that is, (α′*h*v)^2^ vs photon energy (*h*v), for the synthesized samples, were between 3.65 eV (ZLT) and 2.22 eV (ZgP_ZLT_25), which suggests that the combination of silver phosphate with the zeolites Analcime and Pitiglianoite caused the modification of the optical properties. Therefore, obtaining hybrid materials with optical properties distinct from those of pure materials reveals promising characteristics in applications involving fields such as photocatalysis, antimicrobial development, semiconductor properties, and photoluminescent applications.

As can be seen in the curves present in the graphs in Figure 6a,b,e,f, it is observed that the occurrence of only one region for a straight section of the curve indicates that for samples AgP_ZLT_75 and AgP_ZLT_95, there is a predominance of the optical characteristics of silver phosphate.^54^ On the other hand, for samples AgP_ZLT_25 and AgP_ZLT_50, it is noted that a straight section is observed, which closely resembles the optical characteristics of zeolitic structures, occurring at energy values higher than 3.0 eV, i.e., in the ultraviolet region, while at energy values below 3.0 eV, where there is a predominance of electron transitions in the visible spectrum region, another area of straight section occurs, which reinforces the idea that electron transitions associated with the structure of silver phosphate are also present.^92^ Thus, it is confirmed that although the active vibrational modes in Raman spectroscopy were not present for the AgP_ZLT_25 and AgP_ZLT_50 samples, silver phosphate is present in the composition of these samples, which results in the optical characteristics and phase composition already described in the XRD analysis.

Similarly, in the study conducted by Du et al.,^22^ composites were obtained by combining silver phosphate with a colloidal suspension of titanium oxide, using the ratio 80% (TiO_2_):20% (Ag_3_PO_4_), obeying the WT/WT relationship, which resulted in the formation of heterostructures with combined optical characteristics, with the absorption of photons in the ultraviolet region, characteristic of the anatase phase of titanium oxide, as well as absorption of photons in the visible region, which is attributed to the electronic transitions of silver phosphate. In addition, it was noted that the combination of silver phosphate with titanium oxide performed better in terms of photocatalytic performance compared to pure materials, indicating the synergistic contribution to the mixture between the structures of the materials.

Additionally, the optical properties of the pure and combined materials were characterized by using colorimetric analysis, as shown in Table 2. This approach basically consists of obtaining the colorimetric parameters associated with the tristimulus coordinates, based on the principle that for a color to be interpreted, there must be an object, a light source, and an observer.^71^ Thus, the colorimetric analysis will make it possible to obtain the direct or indirect primary colors red (R), green (G) and blue (B), which are related to the colorimetric variables a*, which can assume positive values (indicating colors in red tone) or negative (indicating colors in green tone), and b*, which indicates the blue and yellow color component for positive and negative values, respectively. Finally, the values of L* indicate luminosity, where values close to the upper limit (L* = 100) indicate high brightness samples, i.e., white materials. In contrast, values close to the lower limit, i.e., L* = 0, are obtained for samples with low luminosity, for example, materials in a dark tone or close to black or materials that exhibit high opacity. The Hue angle (h*) refers to the vector resulting from the coordinates in three-dimensional space for the RGB primary colors. In this case, for pure colors, i.e., red, yellow, green, and blue, the values of h* are respectively 0, 90, 180, and 270°. As values between the angles for the colors described, it results from the combination of primary colors and can assume values between 0° and 360°. Finally, chroma (C*) is the parameter that evaluates color intensity, which is different from luminosity. On this scale, higher values indicate more vivid colors, while lower values indicate dull colors or a tendency to gray. The mathematical formulas are presented in eqs 10 and 11.

10 $$h{\;}^{*}=\arctan\left(\frac{a{\;}^{*}}{b{\;}^{*}}\right)$$

11 $$C{\;}^{*}=\sqrt{{a{\;}^{*}}^{2}+b^{2}}$$

**Table 2:**
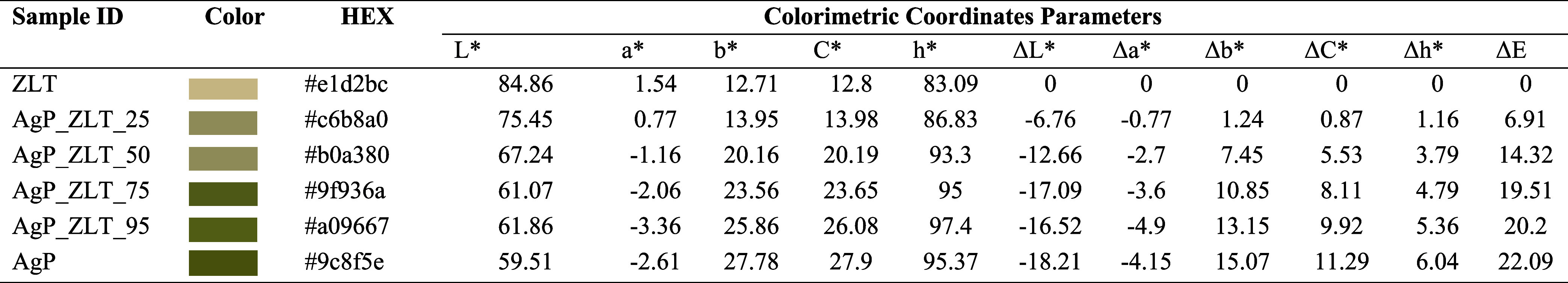
Sample ID, Colorimetric Coordinates Parameters, Color, and Hexacode Index (HEX) of Bare ZLT, Bare AgP, and Hybrid Materials (AgP_ZLT) Prepared at Different Amounts of Silver Phosphate

The difference in color composition (Δ*E*) can be evaluated in terms of the difference in the specific color of a standard matrix in relation to the modification suffered by some physical or chemical factor, which is indicated by the variation perceptible to the human eye. This variation takes into account the square of the variation of the parameters a*, b*, and L*, as mathematically presented in eq 12.

12 $$\Delta E=\sqrt{{\Delta L{\;}^{*}}^{2}+{\Delta a{\;}^{*}}^{2}+{\Delta b{\;}^{*}}^{2}}$$

Based on the results presented in Table 2, the color standard for pure zeolite has a color index of #e1d2bc, indicating colors in light tones with values of L* = 84.86, a* = 1.54, and b* = 12.71, classified as light beige or cream. Because the ZLT sample is the color standard for the matrix, the values of Δa*, Δb*, ΔL*, Δ*C*, and Δ*h* are null. On the other hand, it is noticeable that for pure silver phosphate (AgP), the color index was #9c8f5e, characteristic of materials that provide the olive color, that is, an earthy and soft green-brown tone. The color of silver phosphate can undergo variations, which include the pH, method of synthesis, precursors, heat treatment temperature, crystal size, and morphology. As can be seen from the values of the colorimetric coordinates of CIELab, the values of a* = −2.61 and b* = 27.78 indicate the predominance of the yellow and green components, where the chroma value (C* = 27.9) confirms the relatively vivid color of this inorganic pigment. Among the values obtained for the samples containing the combination of the two materials, i.e., zeolite and silver phosphate in the different proportions studied, it is noted that the sample containing 25% silver phosphate exhibits a predominance of the light beige color pattern, characteristic of zeolite, where the values of the parameters a* and b* are respectively 0.77 and 13.95, However, for samples AgP_LTZ_50 (a* = −1.16 and b* = 20.16), AgP_LTZ_75 (a* = −2.06 and b* = 23.56), and AgP_LTZ_95 (a* = −3.36 and b* = 25.86), it is noted that the values of a* gradually become more negative, i.e., the addition of the yellow tone, which indicates the contribution of electronic transitions related to silver phosphate, This is confirmed by the values of B*, which also gradually increase with the increase of the fraction corresponding to silver phosphate in the composition of the hybrid materials.

Corroborating the information on the variations observed for the colorimetric coordinates of the materials obtained in this study, the values of Δ*E*, available in Table 2, allow us to evaluate the difference between the color of the matrix, i.e., the colorimetric pattern used in this study, the ZLT sample, and the other pigments generated from the mixture of this with different materials. Thus, it is noted that there was a gradual increase in the values of Δ*E* when silver phosphate was introduced into the mixture to obtain the hybrid materials, with respective values of 1.16, 3.79, 4.79, and 5.36 for samples AgP_LTZ_25, AgP_LTZ_50, AgP_LTZ_75, and AgP_LTZ_95. This information supports the other characterization techniques performed, confirming a change in the colorimetric characteristics, which consequently affects the optical properties, as already discussed in the analysis by DRS and XRD spectroscopy.

In the study conducted by Oliveira et al.,^72^ pigments composed of nickel tungstates (NiWO_4_) were efficiently obtained by the method of chemical precipitation followed by heat treatment at 800 °C for 4 h and the method of polymeric precursors, these materials were characterized in detail by colorimetric analysis, correlated to other analytical techniques, where it was possible to observe that the experimental conditions of synthesis led to materials with different colorimetric parameters, and HEX coding. That is, colorimetric variations can occur for the same chemical composition, which can be correlated with oxygen vacancies, the degree of crystallinity, the particle size, and other factors.

The morphology of the crystal structures, investigated by scanning electron microscopy (SEM) and artificially colored using the software Adobe Photoshop CS6, trial version (free availability), for Windows, is presented in Figure 7a–f.

**Figure 7:**
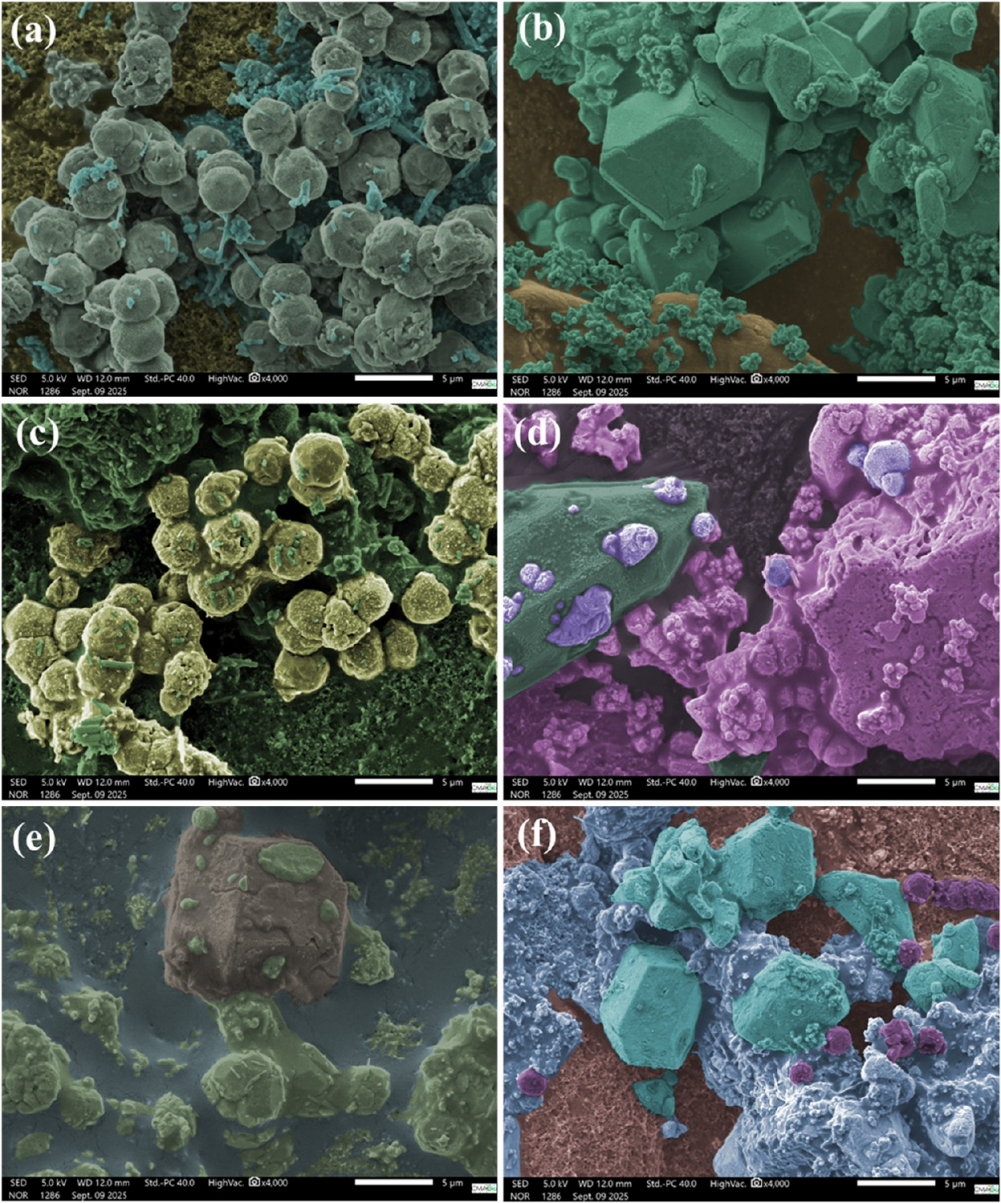
Scanning electron microscopy (SEM) of (a) ZLT, (b) AgP, (c) AgP_ZLT_25, (d) AgP_ZLT_50, (e) AgP_ZLT_75, and (f) AgP_ZLT_95.

As shown in Figure 7a, the Analcime zeolite presents microcrystals with spherical morphology and an average size of 3.25 ± 0.2 μm, resembling the morphology reported by Li et al.,^56^ who obtained microcrystals of the pure and silver-ion-doped Analcime zeolite using the hydrothermal method at a temperature of 155 °C. However, the resulting crystals were close to 10 μm in size. It is also possible to observe the occurrence of microcrystals in the form of elongated rods, with lengths between 0.525 and 3.44 μm, and it is suggested that they are related to the Analcime zeolite phase. As for pure silver phosphate, there was the occurrence of crystals with different morphologies and sizes, as can be seen in Figure 7b, in this case, particles with an approximately spherical shape and an average size of 0.636 ± 0.173 μm, while the larger crystals, with the shape of polyhedrons, presented an average size of approximately 6.98 ± 1.2 μm, that is, heterogeneous behavior, these morphologies being in agreement with those reported in the literature.

From Figure 7c, i.e., sample AgP_ZLT_25, it is possible to observe the occurrence of microcrystals characteristic of both samples, confirming the presence of zeolite in the mixture composition as well as silver phosphate. In several highlights, it is possible to observe spherical crystals, characteristic of ZLT, embedded in the surface of larger crystals (Figure 7d–f), in a polyhedron shape, characteristic of silver phosphate, proving the formation of the heterojunction between the structures and corroborating the other characterization techniques explored in the study. Through transmission electron microscopy analysis (TEM) as can be seen in Figure S4a–d available in the Supporting Information, several silver phosphate nanoparticles are decorating the ZLT surface, increasing the distribution and density of active sites on the hybrid materials.

The semiquantitative analysis of the elements present in the compositions of pure zeolite, pure silver phosphate, and hybrid materials was performed using the energy-dispersive X-ray (EDX), as shown in Figure 8a–f.

**Figure 8:**
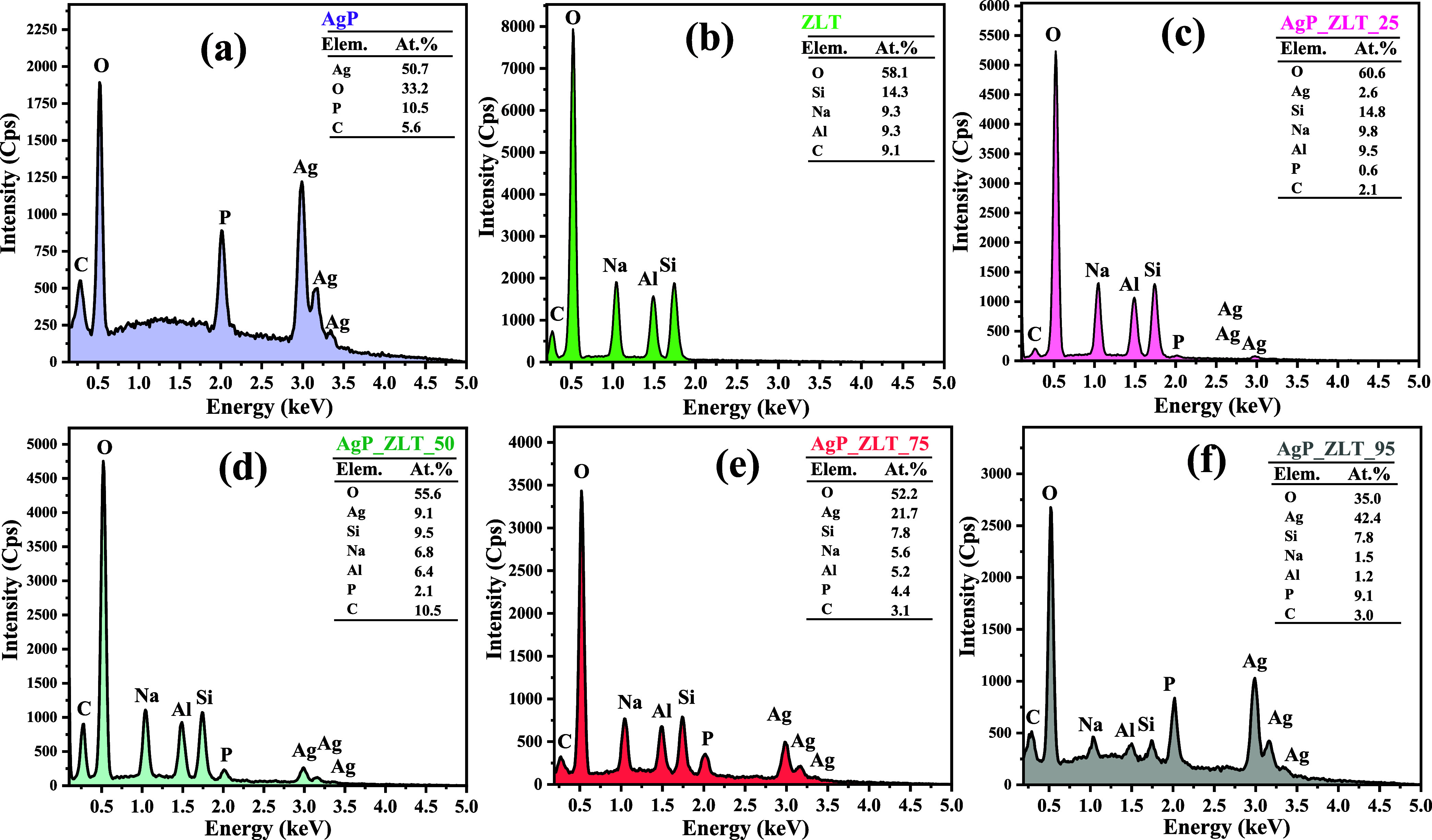
Energy-dispersive X-ray (EDX) for (a) bare AgP, (b) bare ZLT, (c) AgP_ZLT_25, (d) AgP_ZLT_50, (e) AgP_ZLT_75, and (f) AgP_ZLT_95 samples.

Based on the results presented, it is possible to confirm the presence of the elements silver (Ag), oxygen (O), phosphorus (P) and carbon (C) in the AgP sample, confirmed by the indexation of the peaks of dispersive energy (Kα), at 0.29 0.52, and 2.01 keV, respectively, to carbon, oxygen, and phosphorus. In contrast, the peaks associated with the values of 2.97 (Lα), 3.15 (L_β1_), and 3.36 (L_β2_) are characteristic of the element silver. The presence of carbon in the composition of the sample is due to the carbon tape, used to fix the samples on the aluminum metal stubs, which are used during the preparation of the samples, a step that precedes the analysis by EDX.

When performing the analysis of the pure zeolite (ZLT) sample, as shown in the EDX spectrum in Figure 8b, it is noted the presence of dispersive energy peaks in the values of 0.26 keV (Kα), 0.527 keV (Kα), 1.04 keV (Kα), 1.48 keV (Kα), and 1.73 keV (Kα), referring to the elements carbon, oxygen, sodium (Na), aluminum (Al), and silicon (Si), respectively.^66^ All of these are characteristic of the zeolite Analcime, as well as the zeolite Alnacime, which corroborates the discussions carried out in the characterization by X-ray diffraction and structural refinement by the Rietveld method.^97^ Although the crystallographic information on the isomorph of the zeolite Analcime was used, i.e., the crystallographic information on the zeolite Pitiglianoite, the element sulfur (S), which has peak energy dispersion at approximately 2.30 keV (Kα), is absent, which rules out the presence of the zeolite Pitiglianoite. For the spectra of all the other samples, i.e., hybrid samples, as shown in Figure 8c–f, there was the emergence of the peaks of dispersive energy characteristic of the elements present in the silver phosphate matrix, as well as the characteristic elements of the zeolitic support, which reinforces the discussions about the structural, vibrational and optical characteristics already carried out, confirming that for all the samples prepared, there was the presence of both crystalline phases of the oxides of interest.

The percentages of the elements present in the matrix, although estimated by a semiquantitative technique that has limitations, make it possible to affirm the gradual increase in the silver and phosphorus content for the hybrid materials AgP_ZLT_25, AgP_ZLT_50, AgP_ZLT_75, and AgP_ZLT_95, resulting in the respective values of 2.6, 9.1, 21.7, and 42.4%. For the elemental phosphorus, following the same order for the samples, the values were 0.6, 2.1, 4.4, and 9.1%, respectively. Therefore, since the AgP_ZLT_95 sample presents a composition very close to that of the pure sample, AgP, which confirms agreement with the theoretically formulated compositions, this corroborates the analysis of phase composition by structural refinement using the Rietveld method.

The electrochemical properties of the AgP, ZLT, AgP_ZLT_25, AgP_ZLT_50, AgP_ZLT_75, and AgP_ZLT_95 samples were studied by fabricating modified glassy carbon electrodes (GCE), adopting cyclic voltammetry (*C–V*) and electrochemical impedance spectroscopy (EIS) techniques. Thus, potassium hydroxide solution (0.5 mol L^–1^) was used as electrolyte, while silver chloride (Ag/AgCl) and platinum electrodes were used as reference and counter electrodes, respectively. As shown in Figure S5a–d available in the Supporting Information, the potential window for the adopted scan (Figure S5a) was limited to the range of −0.1 and 0.7 V for all cases, adopting a scan rate of 100 mV s^–1^. The results obtained reveal that the standard glassy carbon electrode without active phase coating, i.e., the pure electrode, did not show cathodic or anodic peaks for the oxidative and reductive processes. The same profile was observed for the voltammogram obtained for the electrode containing the ZLT sample, which demonstrates that this pure active system does not have the capacity to intensify the detection signal or undergo oxidative processes in the investigated potential range. In contrast, when the voltammogram was collected for the electrode made with the AgP sample, the appearance of a current peak in the anodic direction was noted, indicating a reduction process. A similar profile was observed by Maraj et al.,^73,74^ where a cathodic peak was identified in the voltammogram of silver phosphate at a potential of 0.41 V, and was attributed to the oxidative process involving silver ions. It is noted that increasing the amount of silver phosphate in combination with zeolite resulted in a considerable increase in the current intensity for the processes involved. For sample AgP_ZLT_50, the cathodic peak appeared near 0.1 V, while samples AgP_ZLT_75 and AgP_ZLT_95 exhibited the highest current intensity for the oxidative processes involved. This suggests that the mixture of materials contributes to electron transfer between the structures, favored by effects such as ion substitution in the zeolite structure as well as crystalline defects and vacancies at the interface between the structures. The graph profile obtained for electrochemical impedance spectroscopy, more specifically the Cole–Cole plot, confirms the information obtained by cyclic voltammetry, where the sample with the lowest curvature for the real (*Z*′) versus imaginary (*Z*″) impedance graph was observed for the AgP_ZLT_95 sample, indicating a shorter relaxation time and, consequently, greater ease in charge transfer. These results are further corroborated by the *Z*′ versus Log frequency and *Z*″ versus Log frequency graphs, which confirm the reduction of *Z*′ and *Z*″ values with the increase in the amount of silver phosphate in the zeolite composition. The AgP_ZLP_75 and AgP_ZLT_95 samples have a graph profile similar to that of pure silver phosphate, suggesting a high ease of electron transport. These characteristics are therefore promising in photocatalytic and antimicrobial processes, facilitating the generation of oxidative radicals or the direct reaction with groups of atoms present in the bacterial cell wall.

The photocatalytic performance of the pure and hybrid materials prepared in this study was investigated using photocatalytic assays with the dye molecule RhB as the standard molecule under blue light of approximately 425 nm wavelength, provided by low-cost and low-consumption light-emitting devices (LEDs). Thus, as can be seen in Figure 9a–h, the spectra of the RhB dye solution at a concentration of 5 mg L^–1^ (ppm) submitted to 10 min radiation exposure tests with a wavelength of 425 nm in the absence (Figure 9a) and presence of pure zeolite (Figure 9b), pure silver phosphate (Figure 9c) and hybrid materials (Figure 9d–g) are presented. as well as the plot of *C*/*C*_0_ versus radiation exposure time in minutes.

**Figure 9:**
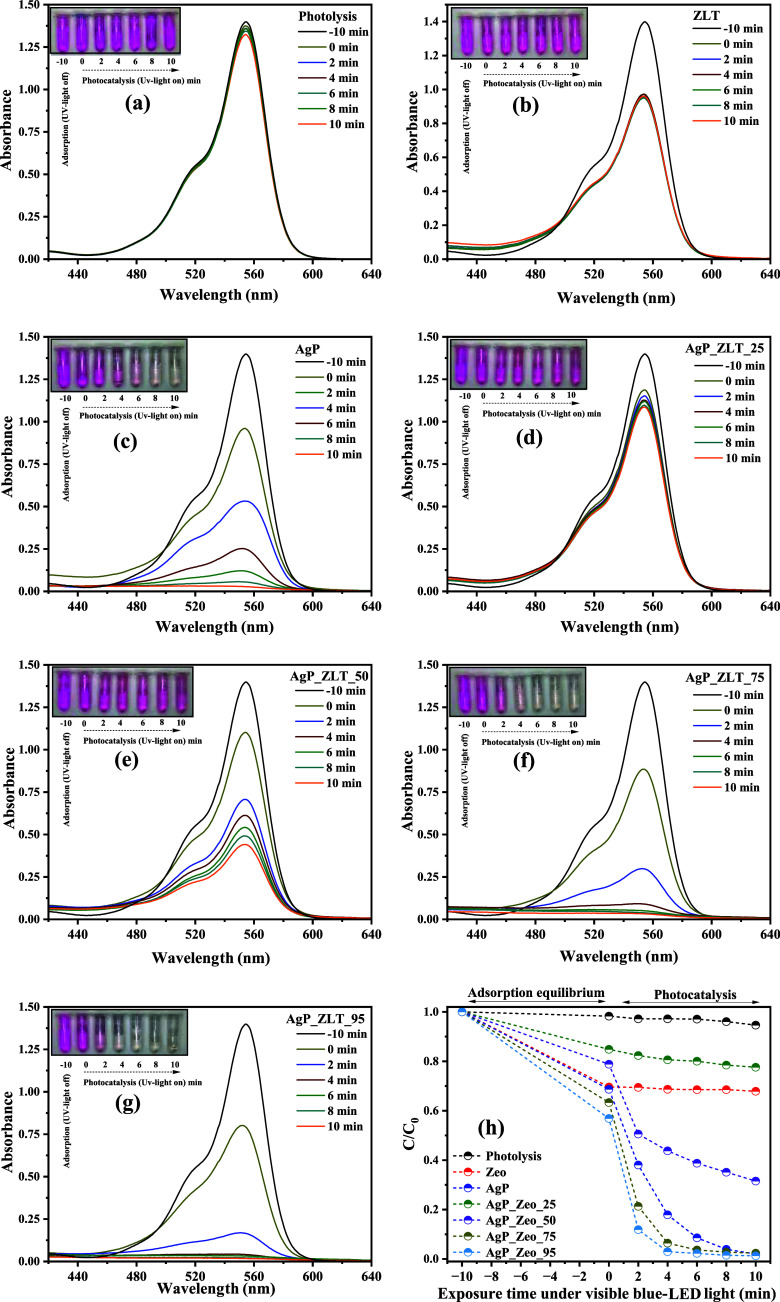
UV–vis spectrum of RhB dye solution at different exposure times under blue LED visible light for (a) photolysis and heterogêneous catalyst, (b) ZLT, (c) AgP, (d) AgP_ZLT_25, (e) AgP_ZLT_50, (f) AgP_ZLT_75, (g) AgP_ZLT_95, and (h) *C*/*C*_0_ against exposure time.

The assay in the absence of the catalyst, commonly called photolysis, resulted in the low degradation performance of the RhB dye, as can be seen in Figure 9a, where the maximum absorption, verified at the wavelength of 554 nm, did not suffer a significant reduction, as a consequence of the photostability of the molecule under the experimental conditions performed. This behavior suggests that once in the environment, the dye molecule RhB exhibits high persistence in physicochemical processes, resulting in reduced light transmittance and biochemical oxygen demand, and consequently compromising the natural activity of aquatic ecosystems.

Figure 9b shows the spectra obtained for the exposure times of the RhB dye solution to radiation using the ZLT sample as photocatalyst, where it is clearly noticeable that after the initial 10 min, i.e., adsorption of the molecules on the catalyst surface under the absence of light, there was a percentage of absorbance reduction of approximately 30%, and that at the end of the radiation exposure time, in this case, 10 min, there was no significant decrease in the absorbance of the dye solution, indicating that the ZLT sample does not present photocatalytic characteristics, only adsorption effect, for the conditions experimentally performed. This behavior can be justified by the value of the prohibited energy band, that is, *E*_gap_, which, through the UV–vis DRS technique, was determined to be equal to 3.65 eV, significantly higher than the energy provided by LEDs, which emit photons with an energy of approximately 2.9 eV. Therefore, it is not sufficient for the excitation of electrons from the valence band to the conduction band, and the consequent formation of the redox pair (h/e^–^), making oxidative processes unfeasible.

In contrast, for the assay performed with pure silver phosphate, rapid discoloration of the RhB dye solution was observed, as shown in Figure 9c. In addition, the graphic profile of the absorbance curves as a function of the wavelength, for the different exposure times. These results made it possible to confirm that at the end of the initial 10 min, in the absence of light, there was a reduction in the absorbance of the RhB dye by approximately 31%, as a result of the electrostatic interaction of the molecules of the RhB dye, which under the conditions presented, behave like cationic molecules on the surface of the catalyst, suggesting, through these observations, that the catalyst has a significant density of negative surface charges. After exposure to light, the crystals of silver phosphate begin to efficiently absorb photons, which is due to the optical characteristics of silver phosphate, resulting in a value of *E*_gap_ equal to 2.35 eV, making it widely favorable for photocatalytic processes under these conditions.

Thus, after 10 min of exposure to visible radiation, there was a percentage of degradation of the RhB dye near 98.72%, when the mathematical expression presented in eq 13 was adopted, where %*D* corresponds to the percentage of degradation, *C*_t_ the concentration of the RhB dye at different exposure times and *C*_0_, the initial concentration of the dye, in this case, 5 mg L^–1^.^75^ Thus, the presence of only 1.28% of the initial concentration of the solution was confirmed, which suggests that this catalyst exhibits an excellent performance in oxidative processes under simulated visible light.

13 $$D\%=\left(1-\frac{C_{t}}{C_{0}}\right)\times 100$$

When the graphic profile for the tests carried out using the hybrid materials in the decolorization of the RhB dye is observed, as shown in Figure 9d–g, it is clearly noticeable that the performance is improved in relation to the results obtained for the ZLT sample, i.e., pure zeolite. In addition, surprisingly, there was a synergistic contribution between the crystalline structures, as can be observed in the results of the AgP_ZLT_75 and AgP_ZLT_95 samples, with the photocatalytic performance being superior to that of pure silver phosphate (AgP) and pure zeolite (ZLT). The improvements also extend to the adsorptive capacity, resulting in increases of 5.7 and 12.1%, respectively, in the AgP_ZLT_75 and AgP_ZLT_95 samples, compared to the AgP sample. When comparing all samples in the time of 4 min and observing the results presented in Figure 9(h), the discoloration percentages follow the decreasing order of performance: AgP_ZLT_95 (*D*% = 97.07%), AgP_ZLT_75 (*D*% = 93.56%), AgP > (*D*% = 82.12%) > AgP_ZLT_50 (*D*% = 56.22%) > AgP_ZLT_25 (*D*% = 19.38%) > ZLT (*D*% = 31.33%) > Photolysis (*D*% = 2.79%).

The kinetic study for the discoloration of the RhB dye molecules was carried out by modeling the data obtained by means of the kinetic models of pseudo-first-order (eq 14)^76^ and pseudo-second-order (eq 15),^77^ as can be seen in Figure 10a,b, as well as the results summarized in Table 3.

14 $$\ln\left(\frac{C_{t}}{C_{0}}\right)=-k_{0}t$$

15 $$\frac{1}{C_{t}}=-k_{0}^{\prime }t+\frac{1}{C_{0}}$$

**Figure 10:**
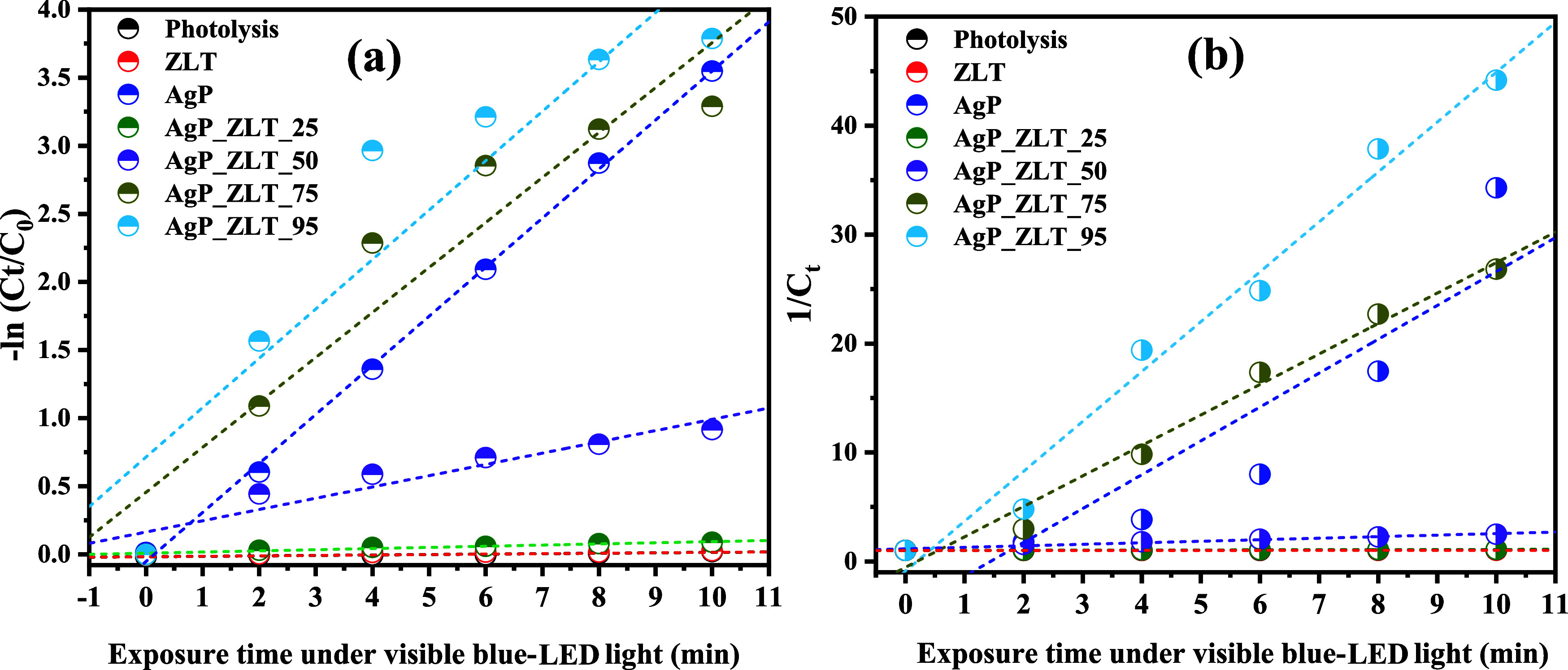
(a) −Ln­(*C*_t_/*C*_0_) against exposure time (min) and (b) 1/*C*_t_ against exposure time (min) for the photocatalytic degradation of RhB dye by bare ZLT, bare AgP, and AgP_ZLT composites.

**Table 3:** Photocatalytic Experiment Results for Photolysis and Heterogeneous Photocatalysis Using ZLT, AgP, AgP, AgP_ZLT_25, AgP_ZLT_50, AgP_ZLT_75, and AgP_ZLT_95 as Solid Catalyst in the Degradation of RhB Dye

	**samples**						
**parameters**	photolysis	ZLT	AgP	AgP_ZLT_25	AgP_ZLT_50	AgP_ZLT_75	AgP_ZLT_95
adsorption (%)^a^		30.4	31.33	15.16	21.17	36.7	43.13
disc. (%)^a^	2.79	31.33	82.12	19.38	56.22	93.56	97.07
*k*_0_ (fisrt-order) × 10^–3^ (min^–1^)	3.23	3.23	360.2	8.52	82.78	330.2	362.7
*r* ^2^	0.8348	0.8348	0.9984	0.9551	0.8670	0.8732	0.8173
*t*_1/2_ × 10^–3^ (min)	214.5	214.5	1.92	81.30	8.37	2.10	1.91
*k*_0_′ (second-order) × 10^–3^ (mol^–1^s^–1^)	3.29	3.29	3108.1	8.93	139.8	2797.1	4578.2
*r* ^2^	0.8319	0.8319	0.7698	0.9604	0.9540	0.9782	0.9769
*t*_1/2_ × 10^–4^ (min)	3039.5	3039.5	3.21	1119.8	71.5	3.57	2.18

a Legend: At time of 4 min.

Based on the results obtained, it is possible to notice that the graphical profile displayed for the linearization of the −ln data­(*C*_t_/*C*_0_) as a function of time, it resulted in a good agreement for the reactions conducted in the absence of catalyst (photolysis), as well as, using the AgP, ZLT, AgP_ZLT_50, and AgP_ZLT25; however, it did not result in a good fit for the data obtained for the AgP_ZLT_75 and AgP_ZLT_95 samples, as can be seen in the values of *r* ^2^, available in Table 3. This kinetic model is widely adopted in the literature^68,70,78,79^ for photocatalytic reactions, especially for reactions that use semiconductors as photoactive materials, where the speed of the reaction is proportional to the concentration of a reactant. While the reactions with second-order kinetics are less reported, they are no less important, and the predominance of the interaction processes between the dye molecules and the active sites available on the catalyst surface.^99^

Therefore, when the second-order kinetics model is adopted, as graphically presented in Figure 10b, it is clearly noticeable that there was a better correlation of the experimental values with the linear adjustment performed, confirmed by the values of *r* ^2^, especially for samples AgP_ZLT_75, AgP_ZLT_75, and AgP_ZLT_95, which presented *r* ^2^ = 0.9540, 0.9782, and 0.9769 (4578.2 min^–1^), respectively. Thus, the percentage of adsorption follows the order: AgP_ZLT_95 (ads = 43.13%) > AgP_ZLT_75 (ads = 36.7%) > AgP (ads = 31.33%) > ZLT (ads = 30.4%) > AgP_ZLT_50 (ads = 21.17%) > AgP_ZLT_25 (ads = 15.16%). While the observed trend for the percentage of discoloration follows the following descending order: AgP_ZLT_95 (*D*% = 97.07%) > AgP_ZLT_75 (*D*% = 93.56%) > AgP (*D*% = 82.12%) > AgP_ZLT_50 (*D*% = 56.22%) > ZLT (*D*% = 31.33%) > AgP_ZLT_25 (*D*% = 19.38%) > Photolysis (*D*% = 2.79%).

The analysis of the results also allows us to infer about the velocity constants for the reactions, in which it is noted that for the experiment in the absence of catalyst (photolysis), AgP, ZLT, and AgP_ZLT_25, there was a better fit of the experimental and modeled results, when the pseudo-first-order model was adopted, while for the other experiments, that is, for the AgP_ZLT_95, AgP_ZLT_75, and AgP_ZLT_50 samples, the fit using the pseudo-second-order model was more evident. In addition, by the values of the velocity constant, it is possible to verify that the photocatalytic experiment performed with the AgP_ZLT_95 sample is about 112.30 times more efficient than the experiment in the absence of the catalyst when the pseudo-first-order model is adopted. On the other hand, when the pseudo-second-order model is adopted, this same ratio reaches a value of 1391.5% more efficient. Thus, it is confirmed that the hybrid catalyst AgP_ZLT_95 presented a synergistic effect for the photocatalytic performance of the RhB dye compared with pure AgP and ZLT precursors.

The half-life time calculated for the photocatalytic reactions performed, in this case, using the pseudo-first-order and pseudo-second-order kinetic models, was estimated using eqs 16 and 17, respectively. As shown in Table 3, the half-life time for the experiment in the absence of the catalyst resulted in a value of 214.5 × 10^–3^ min, which is similar to that obtained for the experiment conducted in the presence of the LTZ sample as a heterogeneous photocatalyst. This behavior indicates that pure zeolite does not exhibit a catalytic profile under the experimental conditions adopted, behaving only as a material with a relative adsorption capacity for the RhB dye molecules. On the other hand, for the reaction conducted in the presence of pure phosphate and silver (AgP), the half-life was only 1.92 × 10^–3^ min, implying that the process takes place around 110.7 times faster. For the hybrid materials, i.e., the samples AgP_ZLT_25, AgP_ZLT_50, AgP_ZLT_75, and AgP_ZLT_95, the half-life times were respectively 81.30 × 10^–3^, 8.37 × 10^–3^, 12.10 × 10^–3^, and 1.91 × 10^–3^ min.


16 $$t_{1/2}=\frac{\ln0.5}{k_{0}}$$

17 $$t_{1/2}=\frac{1}{k_{0}^{\prime }C_{0}}$$

On the other hand, when adopting the pseudo-second-order model, the halfway time for photolysis was 3039.5 × 10^–4^ min, equivalent to that obtained for the experiment with the ZLT sample, while for the AgP, it resulted in the value of 3.21 × 10^–4^ min, which makes it possible to state that AgP is about 946.8 times faster than the experiment in the absence of a catalyst, i.e., photolysis. For the hybrid catalysts, the values are 1119.8 × 10^–4^ min (AgP_ZLT_25), 71.5 × 10^–4^ min (AgP_ZLT_50), 3.57 × 10^–4^ min (AgP_ZLT_75), and 2.18 × 10^–4^ min (AgP_ZLT_95). When comparing the AgP_ZLT_95 sample with photolysis, it is noted that the performance is approximately 1394 times faster, which is higher than that obtained for pure silver phosphate and pure zeolite.

Using a system analogous to the one adopted in this study, do Nascimento et al.,^80,81^ realized photocatalytic degradation of RhB dye using pure silver tungstate (Ag_2_WO_4_), as well, ion-doped silver tungstate copper (Ag_2‑*x*_Cu_*x*_WO_4_), which resulted in 91.2% discoloration of the molecules after 120 min of light exposure, with the constant velocity and half-life time of 2.0 × 10^–3^ min and 34.6 min, respectively. Thus, the authors reveal that there was an increase in the speed of oxidative processes by approximately 16.28 times. In the study conducted by Takeno et al.,^8^ microcrystals of silver phosphates were synthesized by different solvent combinations, using the solvothermal method at 120 °C for 12 h, which resulted in silver phosphate with distinct morphologies, colorimetric, optical and photocatalytic properties, which resulted in the silver phosphates synthesized by mixing the solvents distilled water and acetone (50:50, v/v), as well as isopropyl alcohol and distilled water (50:50, v/v), in the best photocatalytic performances in the decolorization of RhB dye solutions, obtaining a speed constant in the order of 426 × 10^–3^ and 356.2 × 10^–3^ min^–1^, respectively.

Figure 11a,b graphically presents the results obtained from the photocatalytic experiments using the AgP_ZLT_75 sample as a catalyst, varying the initial concentration of the RhB dye (Figure 10a,b), using different substances as radical scavengers in the reaction medium.

**Figure 11:**
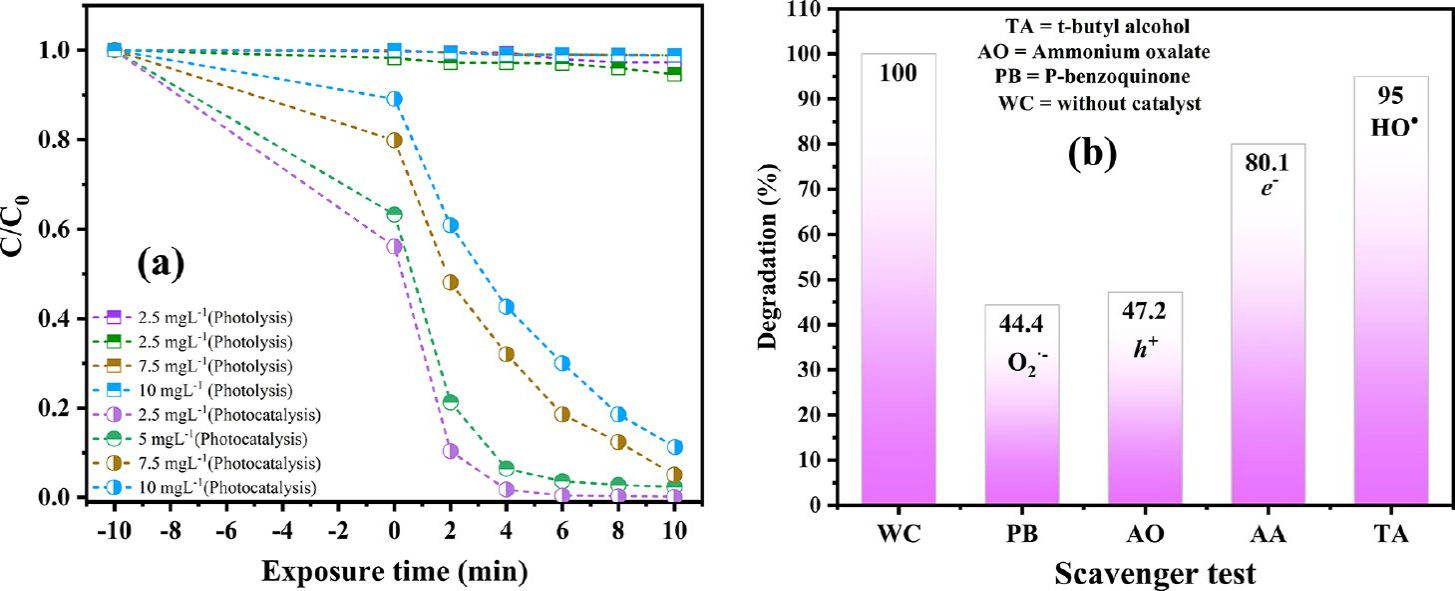
(a) Photocatalytic degradation of different initial concentrations of RhB dye and (b) scavenger’s radicals test for the contribution of each oxidative species in the photodegradation of RhB dye.

As can be seen in Figure 11a, the tests in the absence of the catalyst, i.e., photolysis, using the initial concentrations of 2.5, 5, 7.5, and 10 mg L^–1^, resulted in degradation rates of 5.4, 2.6, 1.2, and 1.1%, respectively. On the other hand, when the catalyst was added to the experiments at the same initial concentration, a significant degradation capacity of the RhB dye molecules was observed, resulting in complete degradation for *C*_0_ = 2.5 mg L^–1^ in just 6 min. For the same exposure time, degradation rates were 6.5, 67.84, and 50.1% for concentrations *C*_0_ = 5, *C*_0_ = 7.5, and *C*_0_ = 10 mg L^–1^, respectively. The reduction in photocatalytic performance when increasing the initial concentration is due to the reduction in photon absorption by the micro- and nanocrystals that make up the catalyst, as well as the saturation of the catalytic sites on the catalyst surface, due to the increased number of molecules in the reaction medium. Similar behavior was observed in the study carried out by Hassani et al.,^78^ where different concentrations of the drug ciprofloxacin were subjected to advanced oxidative processes, using titanium dioxide nanoparticles as a photocatalyst, observing a significant reduction in photocatalytic performance with an increase in the initial concentration of the drug in the reaction medium.

Figure 11b presents the study carried out with the addition of substances that scavenge superoxide radicals (O_2_^*•*–^ (P-benzoquinone, PB)), hydroxyl radicals (HO^•^ (tert-butyl alcohol, TA)), holes (h^+^ (ammonium oxalate, AO)), and electrons (e^–^ (ascorbic acid, AA)).^82^ Therefore, the reduction in photocatalytic performance when each of the substances evaluated is added separately allows us to infer the main radicals that participate in the oxidation of the RhB dye molecules. Thus, as observed, the order of reduction in photocatalytic performance compared to the test without scavenging substances (WC) followed the decreasing order of PB > AO > AA > TA. That is, superoxide radicals and holes are the predominant species in the oxidative process. The contribution of electrons, coupled with holes, occurs as they are photogenerated from the valence band to the conduction band. Hole formation and superoxide radical generation occur in parallel through the reduction of oxygen molecules. Finally, these radicals attack the dye chains adsorbed on the catalyst surface, oxidizing them directly through the holes, while superoxide radicals attack the chromophore group and amino groups of the RhB dye, leading to cleavage and bond rupture.

Silver phosphate with a cubic crystal structure exhibits a high capacity to promote electrons present in the valence band (VB), under the strong contribution of the Ag 4d orbitals as well as O 2p and 2p, to the conduction band, respectively. Since the position of the driving band is close to 2.60 on the potential scale versus NHE (Normal Hydrogen Electrode), which favors the excitation of electrons and generation of holes or gaps (h^+^) in VB.^83–85^ Once microcrystals are on the surface, the water molecules are oxidized, resulting in the formation of H^+^ (hydronium) and hydroxyl ions (HO^–^), as well as the oxidation of dye molecules adsorbed on the surface of the crystalline structures. In this way, they cause the rupture of chemical bonds, generating successive colorless byproducts of lower molecular weight. In addition, the hydroxyl ions generated also undergo oxidation by the holes, resulting in hydroxyl radicals (HO^•^), which are unstable species, with high activity against the chains of organic compounds, attacking them, especially the chromophore groups, breaking the aromatic rings present, and leading to the discoloration of the solution.^5,86,87^

On the other hand, the electrons excited to CB, under the majority contribution of the s orbitals of the elements silver, phosphorus, and oxygen, are captured by the oxygen molecules dissolved in the reaction medium, reducing them to superoxide radicals (O_2_^*•*–^). These species react readily with ions H^+^, as well as groups of atoms present in the carbon chains of the dye molecules, resulting in the attack and consequent compromise of the stability of the primary structure of the compound, transforming it into colorless molecules of lower molecular weight, in the study conducted by Zeng et al.,^88^ photodegradation of the RhB dye was performed using the catalyst BiOCl/g-C_3_N_4_ under visible light, and monitored the degradation products by liquid chromatography coupled to the mass spectrometer, where it was proposed, in agreement with the results obtained, that the dye molecule RhB undergoes a sequence of reactions of loss of the methyl group present in the nitrogen from amines groups, resulting in compounds with a mass/charge ratio equal to 415, 387, 359, 331, and 166 *m*/*z*, from this last byproduct, the opening of the aromatic ring occurs cleavage and subsequent mineralization, that is, formation of gases, as well as inorganic compounds.

The stability of pure silver phosphate (AgP) and heterojunction (AgP_ZLT_75) structures was investigated by using the same catalyst in three consecutive photocatalytic cycles for the degradation of RhB dye molecules in aqueous medium at an initial concentration of 10 mg L^–1^. In this case, at the end of each photocatalytic cycle, the catalyst was collected by centrifugation and then washed with isopropyl alcohol to remove the residual organic fraction adsorbed on the catalyst surface and then reused in a new photocatalytic experiment. Furthermore, the catalyst collected in the last photocatalytic cycle was subjected to analysis by X-ray diffraction and scanning electron microscopy, as shown in Figure 12a–h. The supernatant was subjected to silver determination by inductively coupled plasma optical emission spectrometry (ICP-OES).

**Figure 12:**
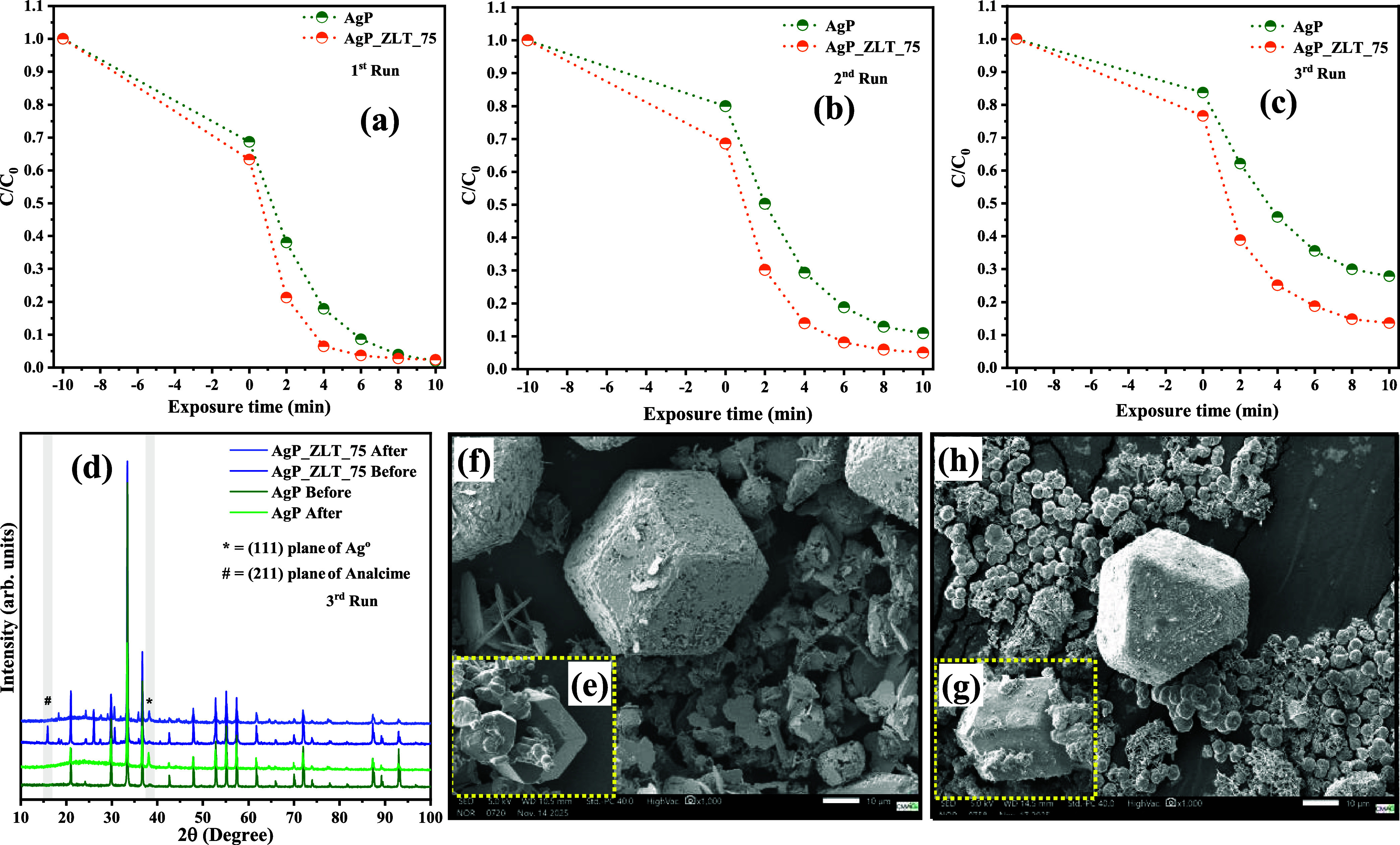
Photocatalytic experiment of reusability for AgP and AgP_ZLT_75 in the (a) first, (b) second, and (c) third cycles. (d) XRD diffraction pattern of AgP and AgP_ZLT_75 before and after the cycling test, and SEM images of AgP and AgP_ZLT_75 before (e, g) and after (f, h) for AgP and AgP_ZLT_75, respectively.

As shown in Figure 12a–c, the AgP sample showed a greater reduction in photocatalytic performance throughout the three photocatalytic assay cycles in the degradation of RhB dye molecules, resulting in percentages of 97.64, 89.07, and 72.11%, respectively. On the other hand, it is confirmed that heterojunction with the zeolite phase mixture resulted in the best performance for all catalytic cycles performed, resulting in percentages of 100, 89.07, and 86.46% for the first, second, and third photocatalytic cycles. When indexing the diffraction pattern of the catalysts before and after the third photocatalytic cycle, it is possible to note that the diffraction pattern for the AgP sample presents, in addition to two characteristic peaks of the cubic structure of silver phosphate, a diffraction peak with average intensity at 2θ = 38.08 and 2θ = 55.05°, associated with the crystallographic planes (111) and (220), which are characteristic of the cubic structure of space group *Fm*3̅*m*, attributed to metallic silver nanoparticles, which perfectly indexed to ICSD card no. 64706 and the consulted literature.^89^ The same occurred with the AgP_ZLT_75 sample; however, in addition to the appearance of the crystallographic plane’s characteristics of silver nanoparticles, there was also a significant reduction of the crystallographic plane (211) at 2θ = 15.99°, associated with Analcime zeolite.

The quantification of metallic silver content (Ag°) in the composition of the phases present was carried out according to the mathematical formalism presented in eq 18, where *A*_(210)_ corresponds to the area of the diffraction peak associated with the cubic structure of silver phosphate, at 2θ = 35.51, while *A*_(111)_ is the area of the diffraction peak at 2θ = 38.08, associated with the crystalline structure of metallic silver nanoparticles.

18 $$X_{{\mathrm{Ag}}^{◦}}=\frac{A_{\left(111\right)}}{A_{\left(111\right)}+A_{\left(211\right)}}\times 100$$

Thus, the phase compositions of the samples AgP and AgP_ZLT are 14.36 and 9.54% for metallic silver, respectively. These results are consistent with the ICP-OES analysis, which resulted in silver content in the supernatant of the solution after the photocatalytic process of 2483.15 and 503.12 ppb for the AgP and AgP_ZLT samples, respectively. Therefore, the combination of silver phosphate with the phase mixture of Analcime and Pitiglianoite zeolites resulted in the preservation of the silver phosphate structure, which acted as a sacrificial structure, preventing photocorrosion of the cubic structure of Ag_3_PO_4_. These statements were confirmed by morphological analysis of the catalysts after reuse in photocatalytic cycles, where it is possible to observe that before the light exposure process, the surface of the silver phosphate microcrystals presents a lower degree of roughness (Figure 12e), holes, or surface defects. However, it is clearly observed that microcrystals undergo photodecomposition (Figure 12f), resulting from a process analogous to the scaling of mesostructures in plates with reduced dimensions, which characterizes the compromise of the primary structure of the catalyst. Although silver leaching occurred in the AgP_ZLT_75 sample, this process occurred under a lower corrosion rate, resulting in less damage to the surface of the characteristic microstructures of Ag_3_PO_4_, which makes it possible to suggest that the reduction in the intensity of the crystallographic plane (211) of the Analcime zeolite is a strong indication of the suppression of the photocorrosion process, as can be seen in Figure 12g,h.

The antimicrobial performance was also investigated for all samples prepared, using the serial microdilution method, which consists of the analysis of the absorbance of the bacteria or fungi suspension. In this study, Gram-positive and Gram-negative multidrug-resistant human pathogen strains and the fungi strains were also investigated.^90–92^ Thus, the fungi *Candida albicans* (*C. albicans*) and *Candida parapsilosis* (*C. parapsilosis*) strains and bacterial *Staphylococcus aureus* (*S. aureus*) and *Escherichia coli* (*E. coli*) strains were tested, according to the graphs available in Figures 13a–l and 14a–l, respectively. The photographic captures of the 96-well plates, used in the microdilution tests, Figure S6, are available in the Supporting Information.

**Figure 13:**
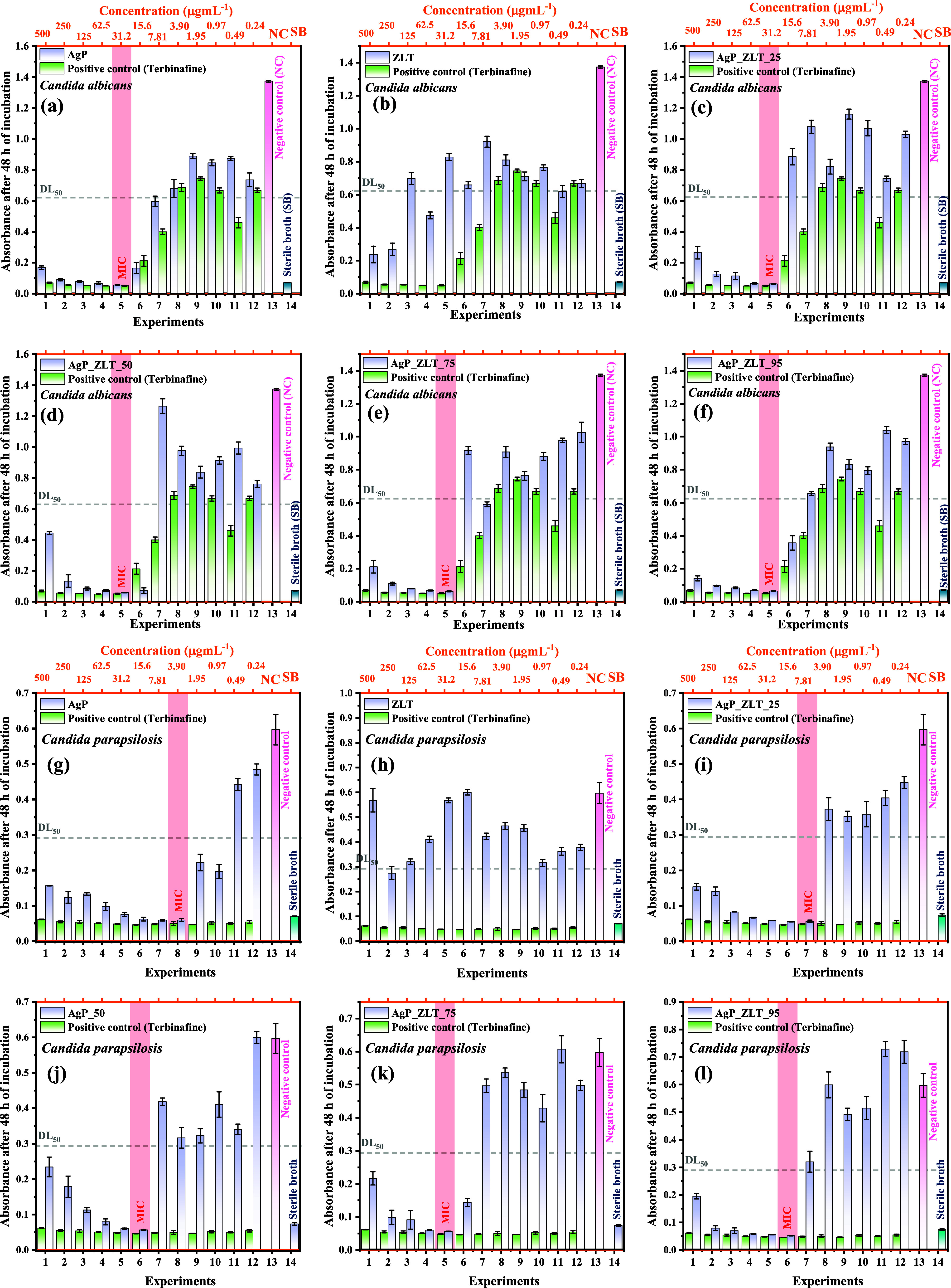
Antimicrobial assay to MIC determination against *C. albicans* (a–f) and *C. parapsilosis* (g–l) using the AgP, ZLT, AgP_ZLT_25, AgP_ZLT_50, AgP_ZLT_75, and AgP_ZLT_95 as biocide agents. The positive control is the Terbinafine drug, while the negative control is the Sabouraud broth.

**Figure 14:**
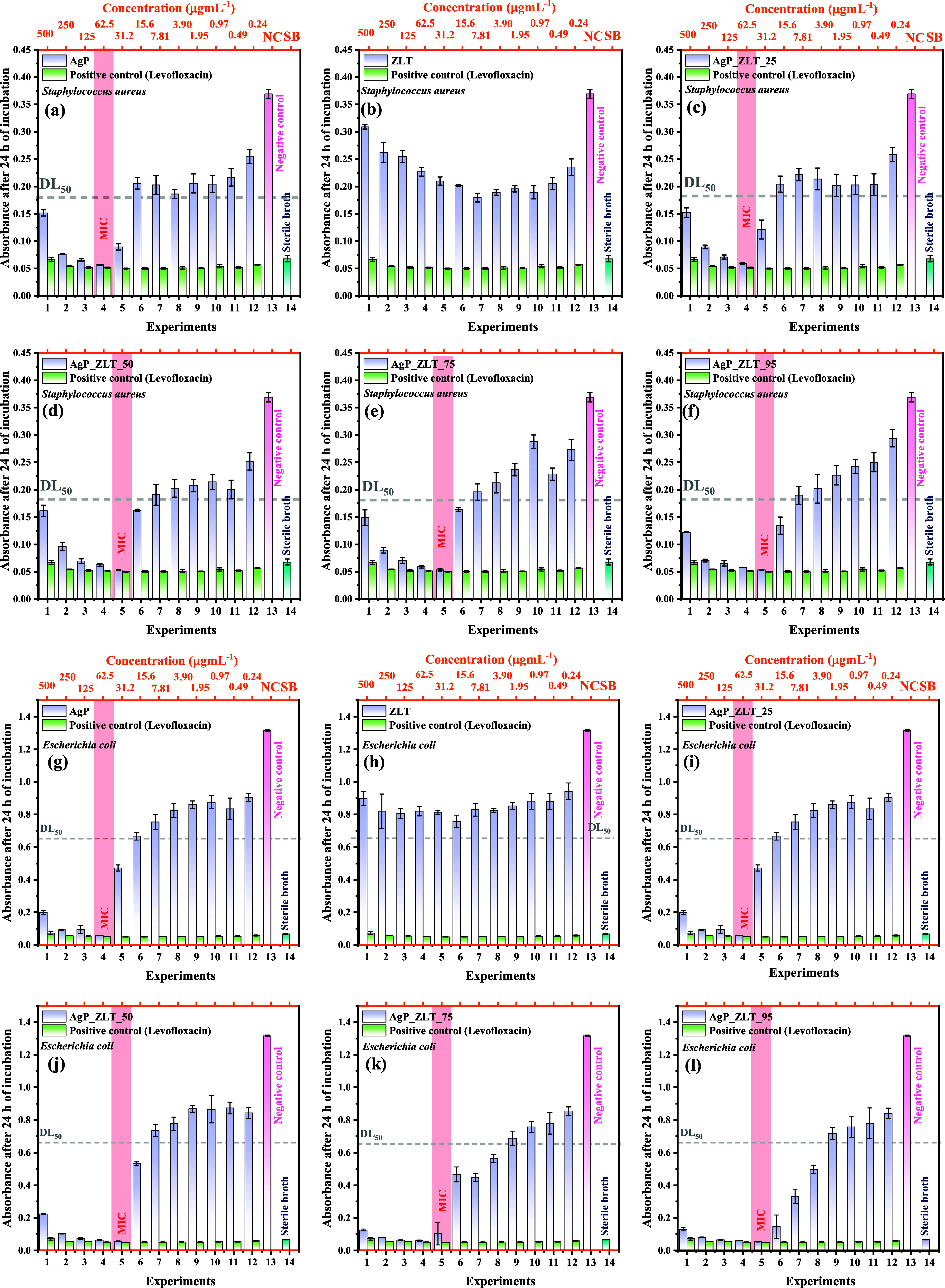
Antimicrobial assay to MIC determination against *S. aureus* (a–f) and *E. coli* (g–l) using the AgP, ZLT, AgP_ZLT_25, AgP_ZLT_50, AgP_ZLT_75, and AgP_ZLT_95 as biocide agents. The positive control is the Terbinafine drug, while the negative control is the Müeller Hinton broth.

Based on the graphs presented for the results obtained in triplicate for each assay, it is noted that silver phosphate, as corroborated by the literature,^7,15,41^ exhibits high antimicrobial performance due to the redox processes provided by silver ions against microorganisms.

In this study, the minimum inhibitory concentrations (MICs) identified by the comparison of the sterile broth absorbance values for the AgP sample obtained for the microorganisms *C. albicans*, *C. parapsilosis*, *E. coli*, and *S. aureus* are 31.2, 3.90, 62.5, and 62.5 μgmL^–1^, respectively. However, the experiments conducted with the ZLT sample exhibited absorbance values lower than the absorbance for lethal concentration (DL_50_), that is, the half-absorbance observed for the negative control is not observed in the MIC values for all tested microorganisms for the range of concentration tested (500 to 0.24 μgmL^–1^), which confirms the low or absence of the antimicrobial activity.

Differently, for AgP_ZLT_25, AgP_ZLT_50, AgP_ZLT_75, and AgP_ZLT_95 samples, there was an increase in antimicrobial performance compared with the ZLT sample. Therefore, the antimicrobial activity order for *C. albicans* was 31.2 μgmL^–1^ for AgP, AgP_ZLT_25, AgP_ZLT_50, AgP_ZLT_75, and AgP_ZLT_95 samples, while for *C. parapsilosis* the MIC values are 15.6 μgmL^–1^ for AgP_ZLT_50, AgP_ZLT_75, and AgP_ZLT_95, 7.81 μgmL^–1^ for AgP_ZLT_95, and 3.90 μgmL^–1^ for AgP sample. For bacteria strains, first, *E. coli*, the MIC value is 62.5 μgmL^–1^ for AgP and AgP_ZLT_25 samples, while for AgP_ZLT_50, AgP_ZLT_75, and AgP_ZLT_95, it was 31.2 μgmL^–1^. Finally, for *S. aureus* strain, the MIC value is 63.5 μgmL^–1^ for AgP and AgP_ZLT samples, while for AgP_ZLT_50, AgP_ZLT_75, and AgP_ZLT_95, it was 31.2 μgmL^–1^.

The positive control for bacteria, in this case, the drug Levofloxacin drug and positive control for fungi strain (Terbinafine drug), resulted in an MIC value less than 0.25 μgmL^–1^ for both bacterial strains and tested fungi strain, which is classified as a high performance.^93^ The molecular dynamics of a drug are entirely different, especially when the solubility coefficient, the presence of specific interaction groups, and the ease of changing the molecule’s conformation are taken into account.^94^ On the other hand, inorganic compounds are crystals with morphology, size, and solubility coefficient that hinder homogeneous dispersion in the reaction environment, depending in most cases on the dynamics of the microorganisms to be able to act against the cell wall.^95^

By comparing the results obtained in this study with those reported by Ibrahim et al.^96^ and Nascimento et al.,^71^ for the same class of microorganisms, it is noted that the hybrid materials prepared in this study exhibit high antimicrobial performance, being superior to that presented in the studies mentioned above, which used silver nanoparticles and copper chloride hydroxide as antimicrobial agents. Among the hybrid materials prepared, the AgP_ZLT_75 sample stands out, exhibiting antimicrobial and photocatalytic behavior very similar to that of the AgP sample; however, it contains 25% zeolite in its composition.

In order to investigate the antimicrobial and bacteriostatic performance of the tested materials, a contact time assay of the materials in the presence of the strains at the concentrations (MIC) previously obtained as described in the preceding paragraphs was carried out. In this case, contact times of 0, 1, 3, 6, 12, and 24 h were investigated for the bacterial strains and times of 0, 1, 3, 6, 12, 24, 36, and 48 h for the tested fungal strains. In these assays, the absorbance of the suspensions at a wavelength of 620 nm was monitored, comparing them with the absorbances of the suspensions containing the respective positive control, which were used in the assays to determine the MIC, as well as the absorbance obtained for the negative control and sterile broth assays.

Figures 14a–l and 15a–l show the absorbances associated with different contact times (hours) of the synthesized materials (AgP, ZLT, AgP_ZLT_25, AgP_ZLT_50, AgP_ZLT_75, and AgP_ZLT_95) in the presence of fungal strains (*C. albicans* and *C. parapsilosis*) and bacteria (*E. coli* and *S. aureus*), as well as the results obtained for the negative controls, positive controls (Terbinafine and Levofloxacin), and sterile broth. As can be observed in Figure 15a–l, with the exception of sample ZLT (Figure 15b), which resulted in increased absorbance after 12 h of contact, evidencing the low antifungal performance of the zeolite, all other samples showed absorbance at a wavelength of 620 nm close to that observed for the absorbance of the assays with the sterile broth, thus confirming that the tested materials exhibited antifungal activity and not only fungistatic activity. Furthermore, the increase in absorbance for the negative control confirms the incubation and growth of the tested strains, thus corroborating the information on the inhibition performance of the inocula containing the hybrid materials and positive control.

**Figure 15:**
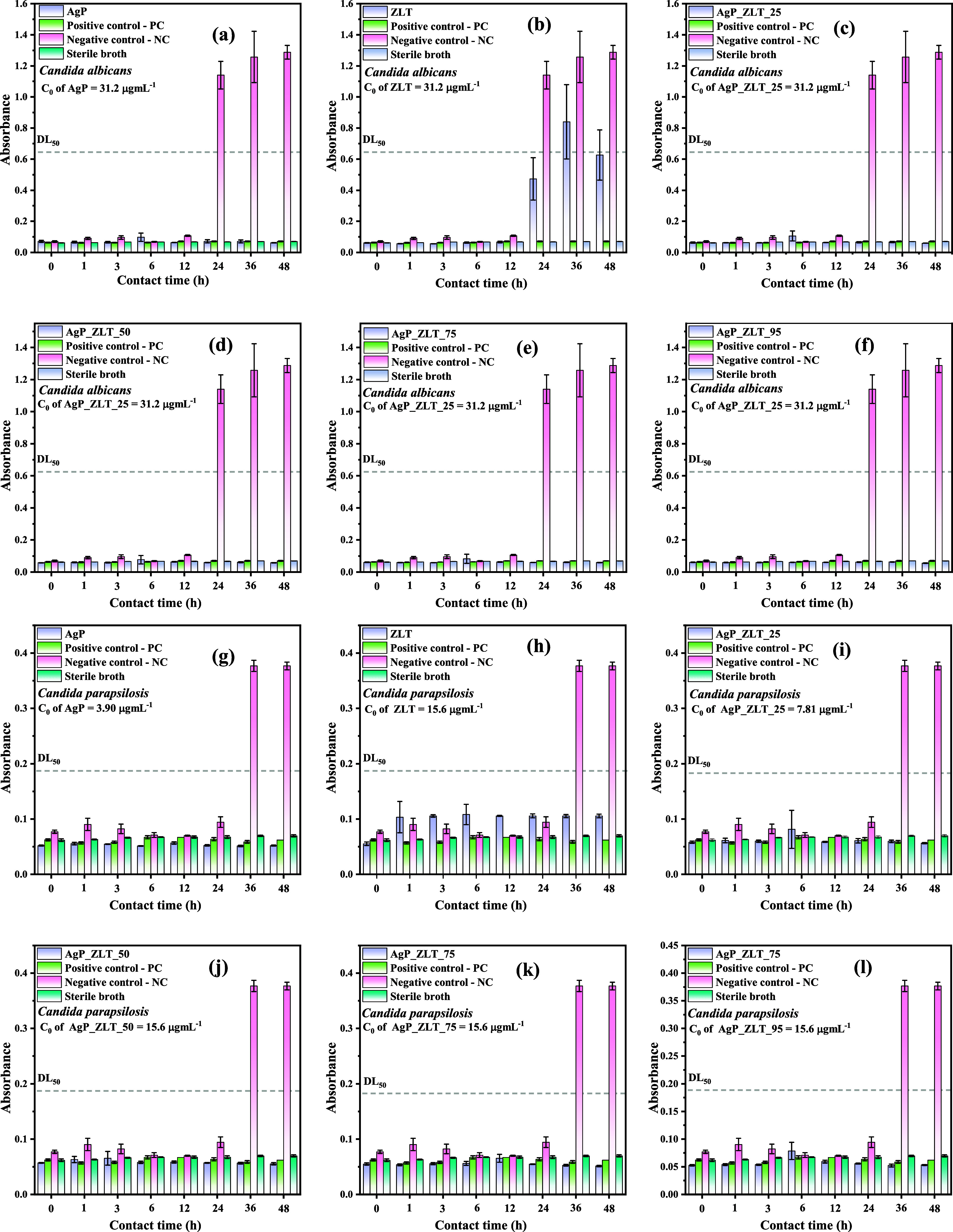
Antimicrobial assay (contact time) against *C. albicans* (a–f) and *C. parapsilosis* (g–l) using the AgP, ZLT, AgP_ZLT_25, AgP_ZLT_50, AgP_ZLT_75, and AgP_ZLT_95 as biocide agents. The positive control is the Terbinafine drug, while the negative control is the Sabouraud broth.

Similarly, the contact time was investigated for *E. coli* and *S. aureus* bacterial strains (Figure 16a–l), using all samples as biocidal agents at the minimum inhibitory concentrations previously determined in the serial microdilution assays described earlier. The positive control was the same as in the previous assays, i.e., Levofloxacin at a concentration of 31.2 μg mL^–1^. Similar to what was observed for the contact time assays with the fungal strains, the ZLT sample showed higher absorbance than the sterile control at a contact time of 12 h, confirming the inability of this compound to control bacteria, with times shorter than 12 h being characteristic of bacteriostatic behavior. On the other hand, the absorbance for all other suspensions that were tested with the samples AgP, AgP_ZLT_25, AgP_ZLT_50, AgP_ZLT_75, and AgP_ZLT_95 showed evident microbiological inhibition, with no microbial growth for the 24 h time interval, similar to the results obtained for the positive control.

**Figure 16:**
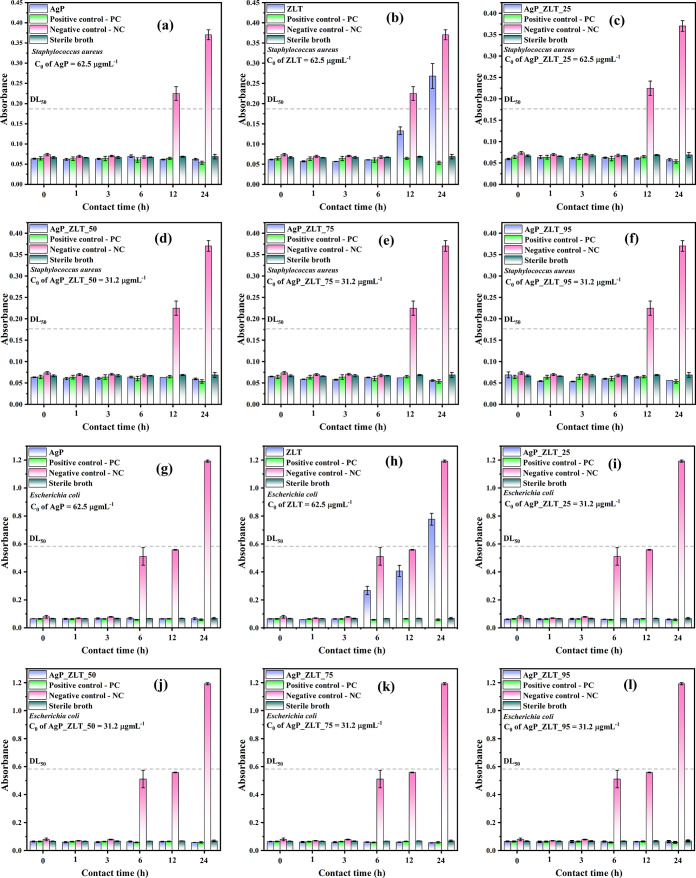
Antimicrobial assay (contact time) against *S. aureus* (a–f) and *E. coli* (g–l) using the AgP, ZLT, AgP_ZLT_25, AgP_ZLT_50, AgP_ZLT_75, and AgP_ZLT_95 as biocide agents. The positive control is the Terbinafine drug, while the negative control is the Müeller Hinton broth.

Based on the information obtained in the photocatalytic and antimicrobial experiments, it was decided to present the schematic representation available in Figure 17, which summarizes the proposed mechanisms involved in the performance exhibited by the AgP, AgP_ZLT_75, and AgP_ZLT_75 samples, which were the two most promising samples among the hybrid materials prepared, for applications in the field of degradation of persistent organic pollutants and antimicrobial agents.

**Figure 17:**
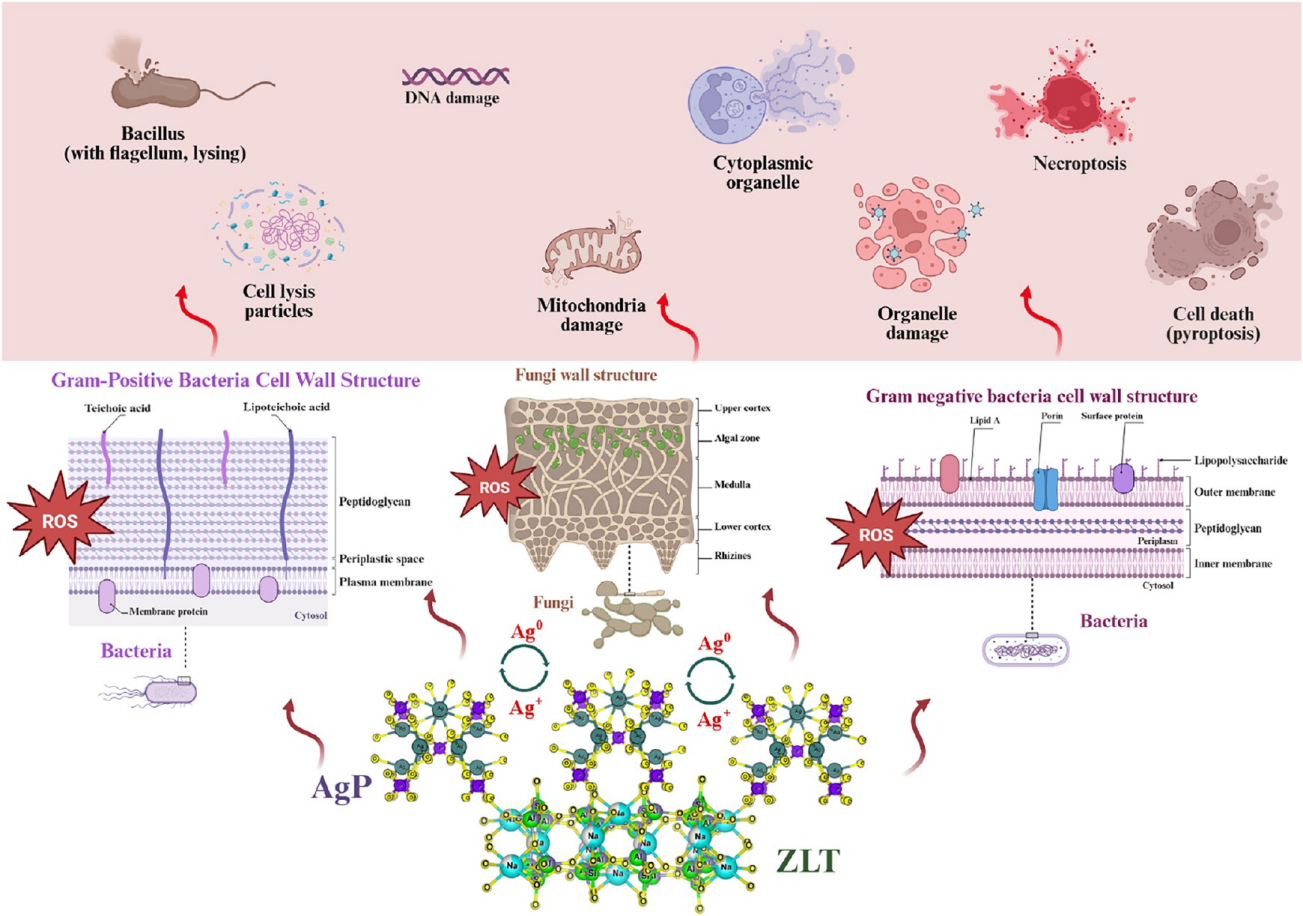
Schematic representation for reactive oxygen species generation from AgP_ZLT heterostructure and Gram-positive, Gram-negative, and fungal cell damage.

As can be seen in the photocatalytic assays, the support used in this study, that is, the mixture between the zeolites Analcime and Pitiglianoite, represented by its isomorph, zeolite Pitiglianoite, exhibited notorious adsorptive capacity against the molecules of the dye RhB, which contributed significantly to the interaction of the silver phosphate microcrystals on the surface of the support, as well as the greater interaction with the molecules of the RhB dye. On the other hand, the crystallographic, optical, and vibrational characteristics of the silver phosphate in the matrix of the hybrid solid resulted in the *E*_gap_ of energy between 2.33 and 2.35 eV, which is largely favorable to the photon absorption with a wavelength in the region of the visible spectrum. Thus, they propose that crystalline defects and cluster deformations [AgO_4_] and [PO_4_] along the three-dimensional crystal lattice enable polarization at short and long ranges, generating ordered clusters ([AgO_4_]_o_ and [PO_4_]_o_) and disordered clusters ([AgO_4_]_d_ and [PO_4_]_d_) as presented in eqs 19 and 20.

19 $${\left[{\mathrm{Ag}}_{3}PO_{4}\right]}_{\left(\mathrm{distorted}\right)}\to {\left[{\mathrm{AgO}}_{4}\right]}_{o}^{x}-{\left[{\mathrm{AgO}}_{4}\right]}_{d}^{x}$$

20 $${\left[{\mathrm{Ag}}_{3}PO_{4}\right]}_{\left(\mathrm{distorted}\right)}\to {\left[{\mathrm{PO}}_{4}\right]}_{o}^{x}-{\left[{\mathrm{PO}}_{4}\right]}_{d}^{x}$$

When irradiated by a light source with a wavelength equal to or greater than 2.35 eV, and in this study, a system with LEDs emitting at a wavelength of 425 nm was used, which provides photons with energy close to 2.9 eV, thus resulting in the process of unpairing the electron–hole pair. For symbolically describing the processes involved, Kröger-Vink’s notation will be used to describe the redox processes involved with silver phosphates, as well as the representation of electrons and holes by the symbols ⊖ to exemplify electrons and ⊕ to exemplify the photogenerated holes. Thus, when photons are absorbed, the following processes of unpairing of electrons in the clusters present in the valence band initially occur, as shown in eqs 21 and 22:

21 $${\left[{\mathrm{AgO}}_{4}\right]}_{o}^{x}-{\left[{\mathrm{AgO}}_{4}\right]}_{d}^{x}+{h\nu }_{\left(2.9\;\mathrm{eV}\right)}\to {\left[{\mathrm{AgO}}_{4}\right]}_{o}^{⊕}-{\left[{\mathrm{AgO}}_{4}\right]}_{d}^{⊖}$$

22 $${\left[{\mathrm{PO}}_{4}\right]}_{o}^{x}-{\left[{\mathrm{PO}}_{4}\right]}_{d}^{x}+{h\nu }_{\left(2.9\;\mathrm{eV}\right)}\to {\left[{\mathrm{PO}}_{4}\right]}_{o}^{⊕}-{\left[{\mathrm{PO}}_{4}\right]}_{d}^{⊖}$$

Since the microcrystals are in an aqueous medium, the water molecules that are adsorbed on the surface of the crystalline structures are oxidized to ions H^+^, ions HO^–^, as well as the conversion of ions HO^–^ in hydroxyl radicals (HO^•^), as well as oxidation of the molecules of the RhB dye by the direct activity of the photogenerated holes, which explains the increase in photocatalytic performance with the increase in the adsorption performance of the materials studied. The reactions involved are presented sequentially in eqs 23–28.

23 $${\left[{\mathrm{AgO}}_{4}\right]}_{o}^{⊕}+{H_{2}O}_{\left(\mathrm{adsorbed}\right)}\to {\left[{\mathrm{AgO}}_{4}\right]}_{o}^{x}+H_{\left(\mathrm{aq}\right)}^{+}+{\mathrm{HO}}_{\left(\mathrm{aq}\right)}^{-}$$

24 $${\left[{\mathrm{AgO}}_{4}\right]}_{o}^{⊕}+{{\mathrm{HO}}_{\left(\mathrm{aq}\right)}^{-}\to \mathrm{HO}}_{\left(\mathrm{aq}\right)}^{•}$$

25 $${\left[{\mathrm{AgO}}_{4}\right]}_{o}^{⊕}+{\mathrm{RhB}\;\mathrm{dye}}_{\left(\mathrm{adsorbed}\right)}\to {\left[{\mathrm{AgO}}_{4}\right]}_{o}^{x}+\mathrm{RhB}\;d\mathrm{ye}{\;}^{*}$$

26 $${\left[{\mathrm{PO}}_{4}\right]}_{o}^{⊕}+{H_{2}O}_{\left(\mathrm{adsorbed}\right)}\to {\left[{\mathrm{PO}}_{4}\right]}_{o}^{x}+H_{\left(\mathrm{aq}\right)}^{+}+{\mathrm{HO}}_{\left(\mathrm{aq}\right)}^{-}$$

27 $${\left[{\mathrm{PO}}_{4}\right]}_{o}^{⊕}+{{\mathrm{HO}}_{\left(\mathrm{aq}\right)}^{-}\to \mathrm{HO}}_{\left(\mathrm{aq}\right)}^{•}$$

28 $${\left[{\mathrm{PO}}_{4}\right]}_{o}^{⊕}+{\mathrm{RhB}\;d\mathrm{ye}}_{\left(\mathrm{adsorbed}\right)}\to {\left[{\mathrm{PO}}_{4}\right]}_{o}^{x}+\mathrm{RhB}\;d\mathrm{ye}{\;}^{*}$$

The electrons promoted to the conduction band migrate to the catalyst/aqueous medium interface, where in the presence of oxygen molecules adsorbed on the surface of the catalysts, there is a reduction of these to superoxide radicals, later hydroperoxides, as well as hydrogen peroxide, all these unstable species, which later contribute to the attack on the carbon chains of the RhB dye. The processes described are outlined in eqs 29–32.

29 $${\left[{\mathrm{AgO}}_{4}\right]}_{d}^{⊖}+O_{2\left(\mathrm{adsorberd}\right)}\to {\left[{\mathrm{AgO}}_{4}\right]}_{d}^{x}+O_{2\left(\mathrm{aq}\right)}^{•-}$$

30 $${\left[{\mathrm{PO}}_{4}\right]}_{d}^{⊖}+O_{2\left(\mathrm{adsorberd}\right)}\to {\left[{\mathrm{PO}}_{4}\right]}_{d}^{x}+O_{2\left(\mathrm{aq}\right)}^{•-}$$

31 $$O_{2\left(\mathrm{aq}\right)}^{•-}+H_{\left(\mathrm{aq}\right)}^{+}\to {\mathrm{HO}}_{2\left(\mathrm{aq}\right)}^{•}$$

32 $${2\mathrm{HO}}_{2\left(\mathrm{aq}\right)}^{•}\to H_{2}O_{2\left(\mathrm{aq}\right)}+O_{2\left(\mathrm{aq}\right)}$$

Since all these photogenerated species are found in aqueous medium, together with the molecules of the RhB dye, successive attacks occur on the carbon chains of the RhB dye, destabilizing them (RhB dye*), which culminates in the primary reactions of dehethylation, followed by the breaking of the chromophore group ring and later mineralization into inorganic compounds and gases, as represented in eqs 32–39.

33 $${\mathrm{HO}}_{\left(\mathrm{aq}\right)}^{•}+\mathrm{Rh}B\;\mathrm{dye}\to \mathrm{RhB}\;\mathrm{dye}{\;}^{*}$$

34 $$O_{2\left(\mathrm{aq}\right)}^{•-}+\mathrm{RhB}\;d\mathrm{ye}\to \mathrm{RhB}\;\mathrm{dye}{\;}^{*}$$

35 $$H_{2}O_{2\left(\mathrm{aq}\right)}+\mathrm{RhB}\;d\mathrm{ye}\to \mathrm{RhB}\;d\mathrm{ye}{\;}^{*}$$

36 $${\mathrm{HO}}_{2\left(\mathrm{aq}\right)}^{•}+\mathrm{RhB}\;\mathrm{dye}\to \mathrm{RhB}\;d\mathrm{ye}{\;}^{*}$$

37 $$\mathrm{RhB}\;d\mathrm{ye}{\;}^{*}\to \mathrm{de}‐\mathrm{ethylation}$$

38 $$\mathrm{RhB}\;d\mathrm{ye}{\;}^{*}\to \mathrm{chromopore}\;\mathrm{cl}\mathrm{ea}\mathrm{vage}$$

39 $$\mathrm{RhB}\;d\mathrm{ye}{\;}^{*}\to \mathrm{mineralization}$$

Regarding the antimicrobial activity observed in this study, as reported by Dakal et al.,^40^ the mechanism of microbial inactivation by metallic nanoparticles and metal ions is still the subject of study around the world and the stage of various discussions, and there is no basic rule for the mechanisms involved, however, it is possible to highlight the following factors, which are commonly observed, they are (i) attraction to bacterial cell walls due to opposite surface charges; (ii) membrane instability; (iii) production of reactive oxygen species (ROS); (iv) release of metal ions; and (v) modification of the signaling route.

In the presence of microorganisms (bacteria and fungi), it is suggested that the silver phosphate microcrystals are surrounded by the cellular interface of the pathogens, which initially try to carry out enzymatic activity to phagocytize particles present in the crystalline structures or transfer electrons to an external solid receptor.^41^ This process triggers redox reactions, which provide the opportunity for the leaching of silver ions into the aqueous medium, and which are easily adhered to the surface of the bacterial wall, as well as entering the cell interior through the cell flow pumps, reaching the cytosol and the cytoplasmic organelles, resulting in the breaking of chemical bonds, rupture of the cell wall and interruption of the basic activities of the cell, leading to cell lysis.^97^

Due to the high reactivity of silver ions with certain chemical elements, mainly phosphorus and sulfur elements, which are commonly present in the carbon chains of cytoplasmic organelles and the genetic machinery of microorganisms, they cause the rupture of these carbon chains, leading to the interruption of protein synthesis and gene replication by the denaturation of deoxyribonucleic acid (DNA) molecules.^7^ This behavior can be more or less effective when comparing the morphological characteristics of microorganisms, especially those classified as Gram-positive and Gram-negative bacteria. In this case, Gram-positive bacteria, such as strains of *S. aureus* evaluated in this study, have a thick bacterial wall compared to Gram-negative bacteria, around 20 to 80 nm, which is generally composed of teichoic acids, especially teichoic acid (WTA), lipoteichoic acid (LTA), which contribute to the maintenance of bacterial wall rigidity, as well as help in cell division, adhesion to surfaces and interaction with the immune systems of host organisms. In addition, they are composed of glycoproteins and peptidoglycans, which have N-acetylglucosamine (GlcNAc) and N-acetylmuramic acid (MurNAc) units linked by β-1,4 bonds.^96,98^

On the other hand, Gram-negative bacteria have a thicker bacterial wall structure than Gram-positive bacteria, around 10 nm or smaller, usually composed of lipopolysaccharide (LPS), lipoprotein, peptidoglycan, and protein and porin structures.^90^ The literature has revealed the greater susceptibility of Gram-negative strains to microbiological inhibition processes by metal oxide nanoparticles, as well as metal nanoparticles and metal ions.^94^ In this case, it is observed that Gram-negative bacteria have negative surface charges, which facilitate the interaction and adhesion of metal cations as well as nanoparticles with positive surface charges, thereby favoring direct redox reactions and consequent damage to the cytoplasmic wall and organelles.

Regarding fungal strains, the antifungal activity of silver-based materials is well established in the scientific literature and silver phosphate is a potent antifungal agent that is not highly selective for different morphologies and physiological characteristics of pathogenic strains in humans. The results obtained in this study are in agreement with the antimicrobial performance reported in the study conducted by Oliveira et al.,^99^ which obtained a minimum inhibitory concentration (MIC) close to 2 and 4 mg mL^–1^ for *C. albicans* suspended and biofilm, respectively, in the absence of visible radiation. These values were reduced in the presence of visible light, yielding values of 0.250 and 2 mg mL^–1^. Based on these results, the authors confirm the contribution of light-promoted oxidative processes, which enhance microbial inhibition through oxidative processes promoted by electron/hole unpairing. Based on this support in the literature, it is possible to suggest that the silver phosphate microcrystals supported on zeolite in the different proportions presented in this study confirmed the high antimicrobial performance, as a result of the distribution of silver phosphate in the zeolite matrix, which favors the increase in the availability of active sites, as well as increased advanced oxidative processes which implies the generation of oxidizing radicals and other reactive oxygen species, as observed in photocatalytic assays against RhB dye solutions.^41^

As shown in Table 4, which presents the characteristics, microorganisms, and performance of different materials applied in the microbial inhibition of various strains of Gram-positive and Gram-negative bacteria and fungi, the materials obtained in this study are promising, being active against fungi and bacteria but not showing significant selectivity for the strains studied. Therefore, they become promising candidates, especially the AgP_ZLT_75 sample, which, even with a 25% reduction in silver phosphate content, showed performance equal to or superior to pure silver phosphate, highlighting the synergistic effect between the zeolite structure and silver phosphate.

**Table 4:** Characteristics of the Prepared and Tested Materials in Comparison with the Antimicrobial Performance of Several Semiconductors Reported in the Literature^a^

			**microorganism**					
**ID**	**composition**	**IH (mm)**	**group**	**species**	**MIC μgmL** ^**–1**^	**SM**	**bandgap (eV)**	**refs**
ZLT	Analcime/Pitiglianoite		Gr–	*E. coli*	>500	HC	3.65	TS
ZLT	Analcime/Pitiglianoite		Gr+	*S. aureus*	>500	HC	3.65	TS
ZLT	Analcime/Pitiglianoite		Fungi	*C. albicans*	>500	HC	3.65	TS
ZLT	Analcime/Pitiglianoite		Fungi	*C. parapsilosis*	>500	HC	3.65	TS
AgP	Ag_3_PO_4_		Fungi	*E. coli*	62.5	HC	2.35	TS
AgP	Ag_3_PO_4_		Fungi	*S. aureus*	62.5	HC	2.35	TS
AgP	Ag_3_PO_4_		Fungi	*C. albicans*	31.2	HC	2.35	TS
AgP	Ag_3_PO_4_		Fungi	*C. parapsilosis*	3.90	HC	2.35	TS
AgP_ZLT_75	Ag_3_PO_4_/Zeolite		Fungi	*E. coli*	31.2	HC	2.33	TS
AgP_ZLT_75	Ag_3_PO_4_/Zeolite		Fungi	*S. aureus*	31.2	HC	2.33	TS
AgP_ZLT_75	Ag_3_PO_4_/Zeolite		Fungi	*C. albicans*	31.2	HC	2.33	TS
AgP_ZLT_75	Ag_3_PO_4_/Zeolite		Fungi	*C. parapsilosis*	15.6	HC	2.33	TS
IE	SOD-Ag	7.80	Gr+	*S. aureus*		IE		^101^
CP	Ag_3_PO_4_/MMT		Gr–	*E. coli*	31.25	CP		^59^
CP	Ag_3_PO_4_/MMT		Gr+	*S. aureus*	62.5	CP		^59^
TH	Ag_3_PO_4_		Gr–	*E. coli*	15.62	CP		^15^
TH	Ag_3_PO_4_		Gr–	*Klebsiella planticola*	31.25	CP		^15^
TH	Ag_3_PO_4_		Gr+	*S. aureus*	31.25	CP		^15^
TH	Ag_3_PO_4_		Gr+	*Micrococcus luteus*	15.62	CP		^15^
AgNPs	Ag		Gr+	*S. aureus*	625	BS		^96^
20 min	Cu_2_(OH)_3_Cl		Fungi	*C. albicans*	0.5	CP	3.13	^71^
30 min	Cu_2_(OH)_3_Cl		Fungi	*C. parapsilosis*	0.5	CP	3.09	^71^

a Legend: SM = synthesis method; BS = biosynthesis; IH = inhibition halo; PS = particle size, IE = ion exchange; CP = coprecipitation; MIC = minimum inhibitory concentration; refs = references.

In Figure S4a–d, available in the Supporting Information, it is possible to observe that the AgP_ZLT_75 sample presents nanoparticles with dimensions between 9.42 and 12.5 nm, anchored to the surface of the zeolite structure. This is believed to increase the surface area, thus preventing nanoparticle agglomerates that could precipitate and consequently reduce the contact area with microorganisms. This characteristic is one of the key points for improving antimicrobial and photocatalytic properties.^40,100–103^

## 4 Conclusion

In summary, the phase mixture of zeolite Analcime and Pitiglianoite was achieved using biogenic silica and Amazonian metakaolin by the hydrothermal method. The bare zeolite, bare silver phosphate, and heterostructures were structurally characterized by X-ray diffraction and Rietveld refinement, which provided the opportunity to confirm the percentages of silver phosphate of 7.99 ± 0.46% (AgP_ZLT_25), 36.70% ± 4.84% (AgP_ZLT_50), 67.50 ± 5.06% (AgP_ZLT_75), and 93.74 ± 3.15% (AgP_ZLT_25). The semiquantitative characterization by X-ray dispersive energy (XRD) revealed the presence of dispersive energy peaks associated with all the characteristic elements of zeolite (Na, Si, Al, and O), as well as for pure silver phosphate (Ag, P, and O), these elements being present in the hybrid materials, confirming the gradual increase of the atomic percentage referring to silver and phosphorus, corroborating the other characterization techniques. The photocatalytic performance of the hybrid materials in the discoloration of RhB dye solutions under visible radiation from LEDs with a wavelength of 425 nm confirmed that the AgP_ZLT_95 and AgP_ZLT_75 samples exhibited a high rate of generation of reactive oxygen species (superoxide radicals and holes), higher than that obtained for pure silver phosphate. In particular, the AgP_ZLT_95 sample exhibited a velocity constant, as determined by the pseudo-second-order kinetics model, and a half-life time, which was approximately 1391.50 times more efficient compared to the reaction performed in the absence of a catalyst. The reusability of the catalyst test confirms the synergic effect of the zeolite matrix for silver phosphate, which reduces the photocorrosion of silver clusters close to 5%. Finally, the microbiological assays adopting the serial dilution method against strains of Gram-positive bacteria (*S. aureus*) and Gram-negative (*E. coli*) resulted in the MIC of 31.2 μgmL^–1^, while for the fungi tested, in this case, *C. parapsilosis* and *C. albicans*, the MICs obtained were 15.6 and 31.2 μgmL^–1^. Thus, silver phosphate loaded in zeolite structure exhibits high performance in microbiological inhibition and applications in the photodegradation of persistent organic pollutants.

## Supplementary Material



## Notes

X-ray diffraction (XRD) and elemental X-ray fluorescence (XRF) of biogenic silica obtained from kaolin; diffraction patterns of kaolinite and metakallin; quantification of silver ions in postphotocatalytic experiment solutions by atomic absorption spectroscopy (ICP-OES); transmission electron microscopy (TEM) of silver phosphate and heterojunction; cyclic voltammetry, Cole–Cole, real and imaginary impedance spectroscopy; and photographic captures of the 96-well plates from the antimicrobial experiments (PDF)
