# Supplementary material for: Antimicrobial and Photocatalytic Properties under Visible Blue LED Light of Silver Phosphate Supported on Biogenic Zeolite from Amazon Natural Source
Source: ACS Omega. 2026 Feb 27;11(9):14501–32. doi: 10.1021/acsomega.5c09905 (PMC12980204; doi:10.1021/acsomega.5c09905)
Supplement: Supplementary file 1 [file ao5c09905_si_001.pdf]

Support Information

Antimicrobial and Photocatalytic Properties under visible Blue LED light of Silver Phosphate Supported on biogenic Zeolite from Amazon Natural Source

Ygor Geann dos Santos Leite[1#], Francisco Xavier Nobre[2*#], José Fábio de Lima Nascimento[2], Wesley Victor De Sombra Quércia[2], Raiana Silveira Gurgel[3], Patrícia Melchionna Albuquerque[3], Yurimiler Leyet Ruiz[4], Ézio Sargentini-Júnior[5], Marcos A. Bolson[5], Yonny Romaguera-Barcelay[6], Ramón R. Peña-Garcia[6], Paulo Rogério da Costa Couceiro[7], Rosany Picolotto Carvalho[1]

[1]*Programa Multi-Institucional de Pós-Graduacão em Biotecnologia, Universidade Federal do Amazonas, 69077-000 Manaus, AM, Brazil.*

[2]*Grupo de Recursos Energéticos e Nanomateriais (GREEN), Instituto Federal do Amazonas, Campus Manaus Centro (IFAM-CMC), Avenida Sete de Setembro, 1975, 9020-120, Manaus, AM, Brazil.*

[3]*Research Group on Chemistry Applied to Technology, School of Technology, Amazonas State University, Manaus, 69050-020, Brazil.*

[4]*LPMAT - Department of Materials Engineering, Federal University of Amazonas, 69077-000, Manaus, AM, Brazil*

[5]*Instituto Nacional de Pesquisas da Amazônia (INPA), 69084-000, Manaus, AM, Brazil.*

[6]*Universidade Federal Rural de Pernambuco, Programa de Pós-Graduação em Engenharia Física, Unidade Acadêmica do Cabo de Santo Agostinho, Recife-Pernambuco 54518-430, Brazil.*

[7]*Department of Chemistry, Federal University of Amazonas,69077-000 Manaus, AM, Brazil.*

**Corresponding author: F.X. Nobre (francisco.nobre@ifam.edu.br)*

[#]*These authors contributed equally to this manuscript.*

Chapter Figure

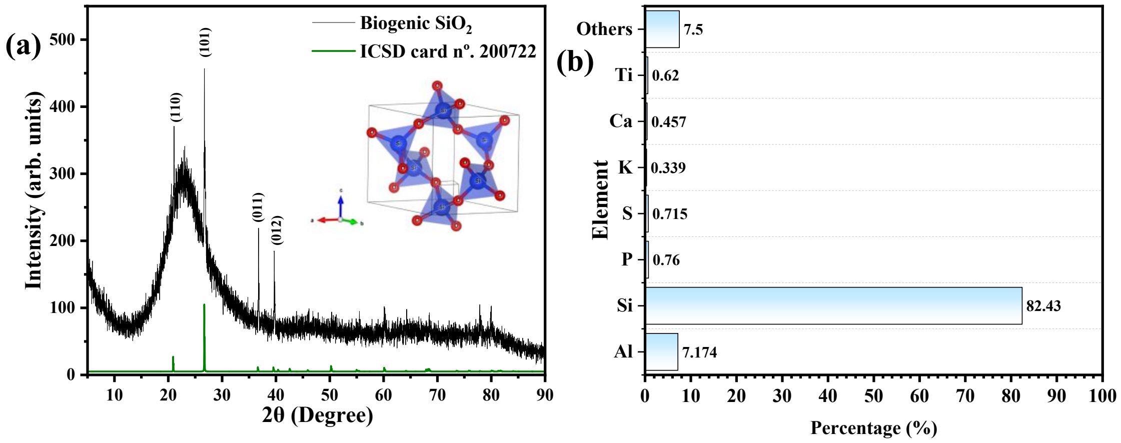

Figure S1: (a) X-ray diffraction pattern and (b) X-ray fluorescence spectroscopy of biogenic SiO_2.

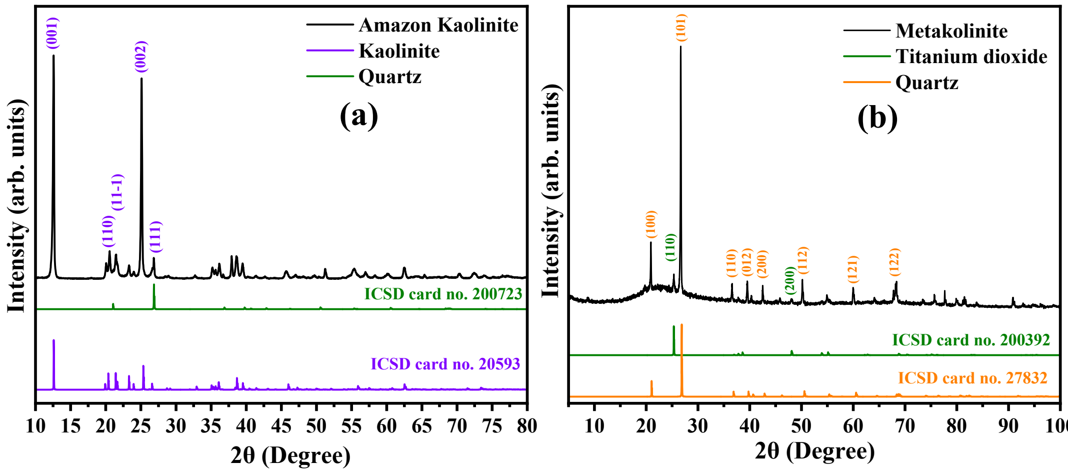

Figure S2: (a) Powder diffraction pattern of natural Kaolinite and (b) Metakaolinite.

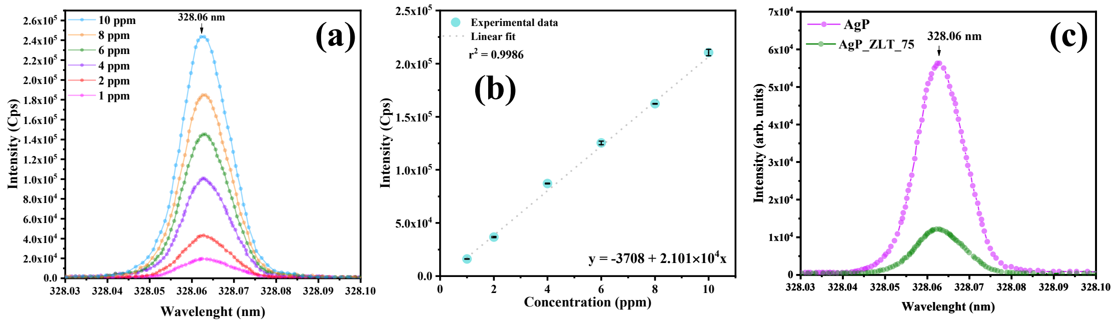

Figure S3: (a) and (b) ICP-OES curve of Ag^{+} ions in the interval from 1 to 10 ppm, and (c) the determination of intensity for supernatant from recycling test in the photodegradation of RhB dye solution for AgP and AgP_ZLT_75 catalyst.

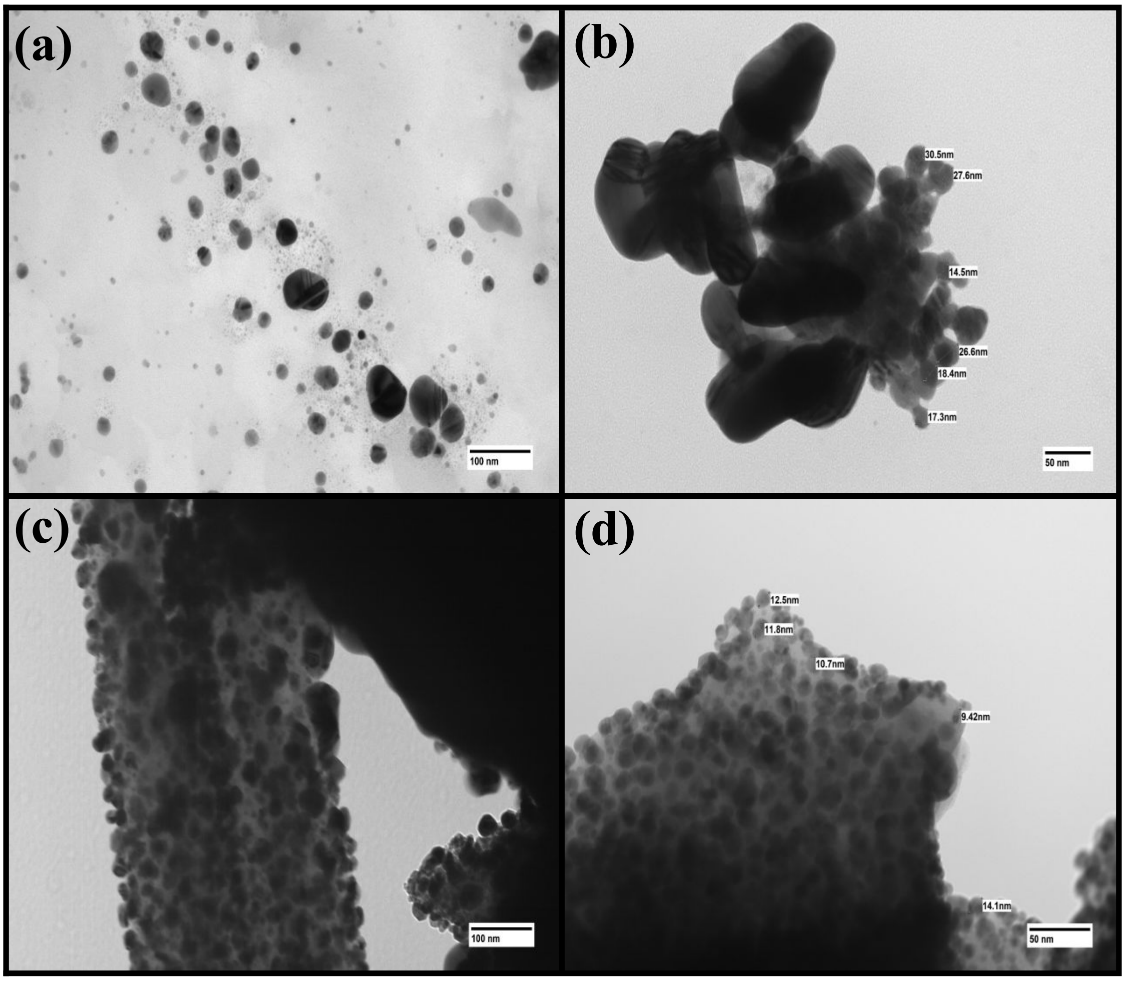

Figure S4: Transmission electron microscopy of (a) and (b) AgP and (c) and (d) AgP_ZLT_95 samples.

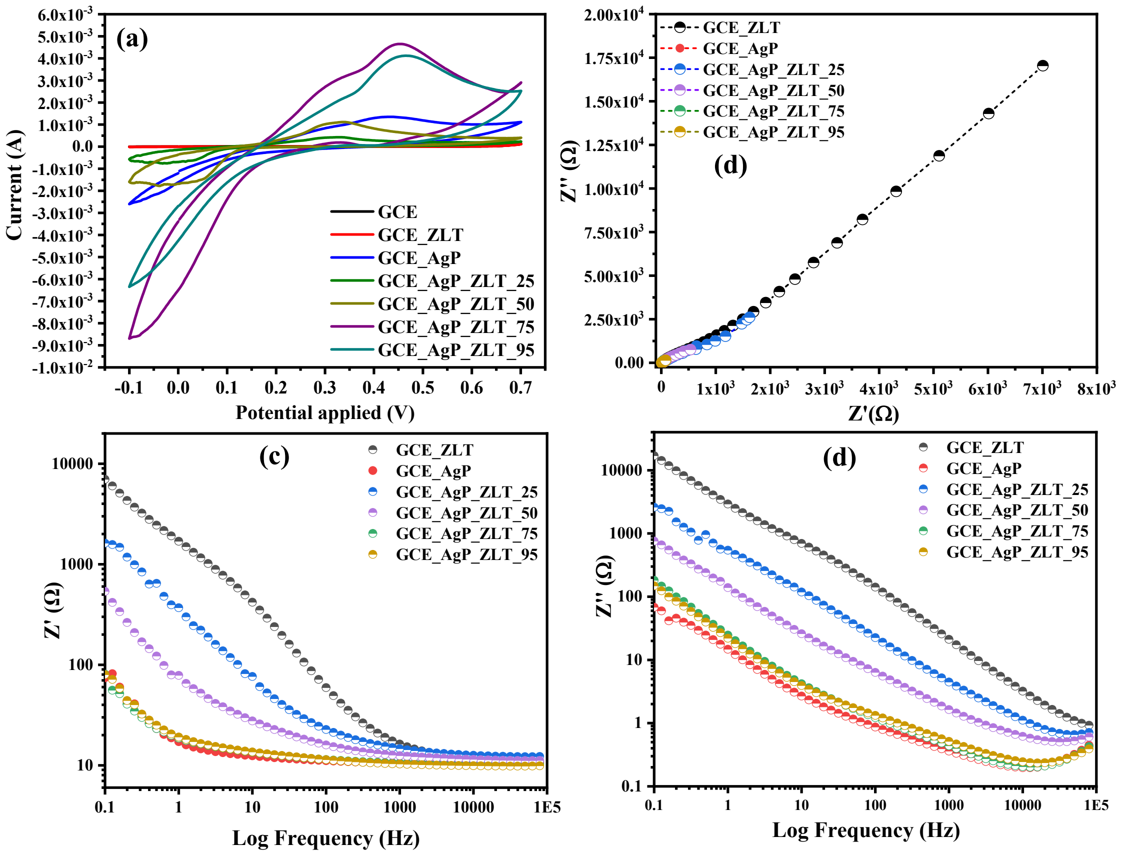

Figure S5: (a) Electrochemical cyclic voltammetry, (b) real versus imaginary (col-cole) plot, (c) real impedance versus log frequency and (d) imaginary impedance against log frequency.

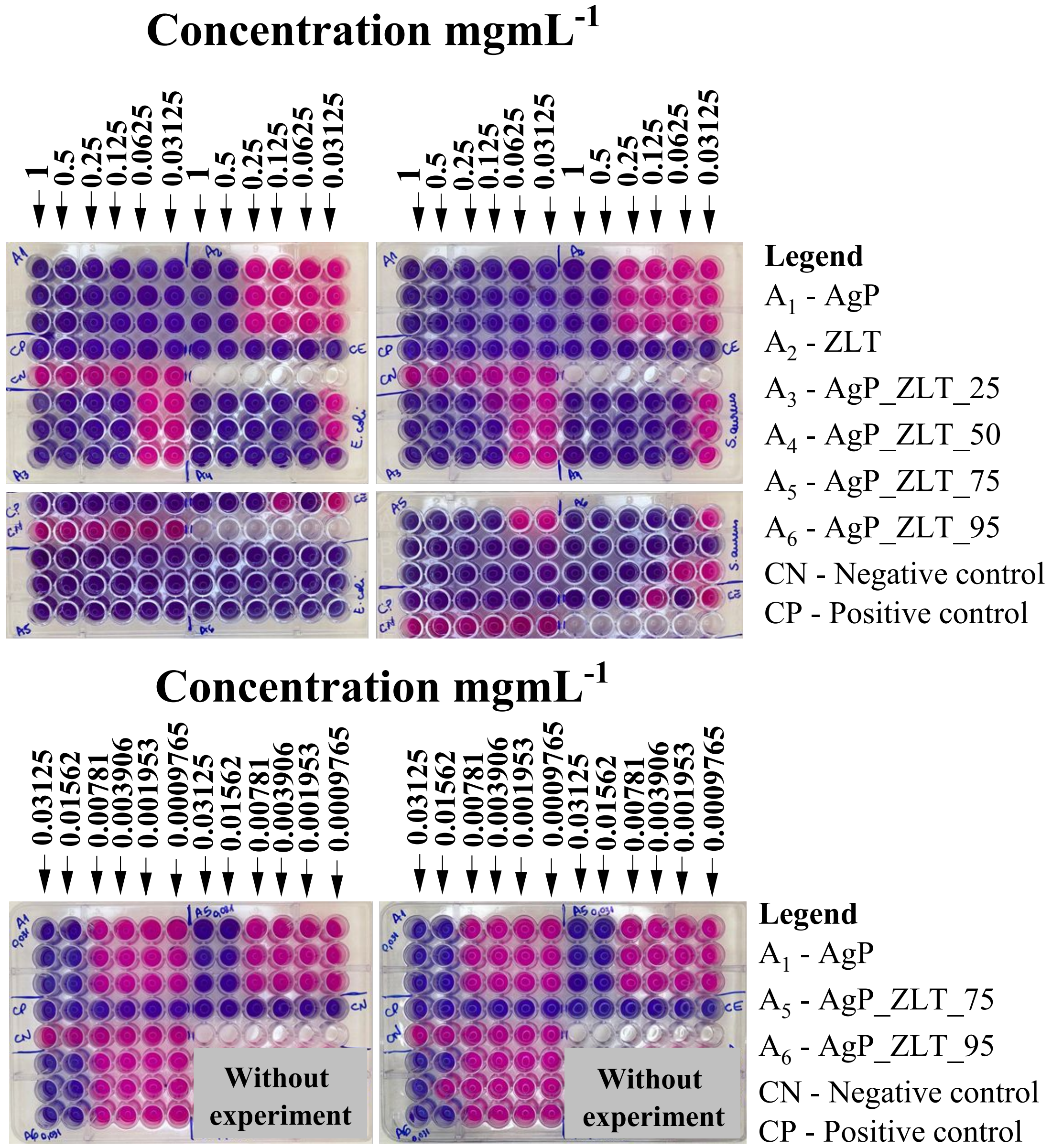

Figure S6: Digital photography of 96-well plate and the results for antimicrobial assay with AgP, ZLT and AgP_ZLT_25, AgP_ZLT_50, AgP_ZLT_75, AgP_ZLT_75 and AgP_ZLT_95 samples.
